# Manipulation of coacervate droplets with an electric field

**DOI:** 10.1073/pnas.2203483119

**Published:** 2022-08-04

**Authors:** Aman Agrawal, Jack F. Douglas, Matthew Tirrell, Alamgir Karim

**Affiliations:** ^a^William A. Brookshire Department of Chemical and Biomolecular Engineering, University of Houston, Houston, TX 77204;; ^b^Materials Science and Engineering Division, National Institute of Standards and Technology, Gaithersburg, MD 20899;; ^c^Pritzker School of Molecular Engineering, University of Chicago, Chicago, IL 60637

**Keywords:** coacervate droplets, polyelectrolytes, droplet stabilization, encapsulation, electrorheological fluid

## Abstract

Many biopolymers are highly charged, and as in the case of many polymer mixtures, they tend to phase separate as a natural consequence of chain connectivity and an associated relatively low entropy of polymer mixing. Recently, it has become appreciated that the phase-separated structures formed by such polyelectrolyte blends, called “complex coacervates,” underlie numerous biological structures and processes essential to living systems, and there has been intense interest in understanding the unique physical features of this type of phase-separation process. In the present work, we are particularly concerned with the field responsiveness of stabilized coacervate droplets formed after the phase separation of polyelectrolyte blend solution and then exposed to deionized water, making the droplet interfacial layer acquire a viscoelastic character that strongly stabilizes it against coalescence. We show that we can precisely control the positions of individual droplets and arrays of them with relatively low-voltage electric fields (on the order of 10 V/cm) and that the imposition of an oscillatory field gives rise to chain formation with coarsening of these chains into long fibers. Such a phase-separation–like process is generally observed in electrorheological fluids of solid colloidal particles subjected to much larger field strengths. The key to these coacervates’ electrorheological properties is the altered interfacial viscoelastic properties when the droplets are introduced into deionized water and the associated high polarizability of the droplets, similar to the properties of many living cells. Since many different molecular payloads can be incorporated into these stable droplets, we anticipate many applications.

## Significance

Many of the unique properties of biological materials are derived from the highly charged nature of the constituent molecules and their diffuse counterion clouds, which often render such materials intrinsically polarizable, and thus highly responsive to electric fields. We investigate a synthetic material of this kind created through the formation of polyelectrolyte coacervates transferred to distilled water to extract excess counterions to form highly stable droplet suspensions in which individual droplets, and their large configurations, can be precisely manipulated with field strengths comparable in magnitude to a 9-volt battery. These materials should be useful in encapsulating, transporting, and delivering various cargos in numerous applications in manufacturing and medicine and as a model system for understanding electrodynamic aspects of living systems.

There has been a long-standing interest in manipulating particles through the application of electric fields to control their individual positions, as well as the large-scale organization of many particles within different materials. Most of this work has been devoted to hard colloidal particles dispersed in solvents, such as in electrorheological (ER) fluids where particles form chains under a high electric field (1–5). However, we know that electrical stimuli direct particle motion and organization in critical biological processes, such as wound healing, where the particles are cells or other “soft” vesicle-like structures (6, 7). We thought it would be of interest to develop field-responsive vesicle-like soft particles that might serve as vehicles for transporting cargos of various types ranging from scents and various reactive species in a manufacturing context related to personal care and food science products to proteins, genetic materials, or synthetic drugs in a medical science context. Such a material system would require several physical attributes to function as a versatile carrier for these diverse cargos and medical applications and must overcome significant additional limitations concerning the stability and biocompatibility of the material utilized. Finally, as in all materials science applications, there are cost issues and the difficulty of the fabrication process that must be considered.

The basic properties that we must have for our field responsive droplets are that they have interfacial viscoelasticity to inhibit their coalescence when they come into close contact with each other, and they must have an appreciable surface charge or high dielectric mismatch with the solvent (aqueous salt solutions in case of a biocompatible system) to make the droplets sensitive to being manipulated individually or collectively by an electric field. There are limitations of the field strength also. In most existing ER fluid applications, the field strengths are far too large (on the order of 1 to 10 kV/cm) to be compatible with the applications just mentioned (2, 4, 5). Clearly, these restrictions greatly constrain the class of materials that might be suitable for this hypothetical type of ER fluid.

Nature has apparently solved this problem evolutionarily by creating phase-separated “membraneless” droplets of nucleic acids and proteins, called “complex coacervates” or “condensates,” which are ubiquitous in the biological world in the form of organelles such as Cajal bodies, P-granules, and many other fundamentally important biological cells structures (8, 9). Correspondingly, a significant amount of recent research has gone into identifying the (bio)physical characteristics (e.g., thermodynamics, rheology, molecular partitioning) of these phase-separated droplets using model coacervate systems of charged polymers, both natural and synthetic, to understand their role in the origin of life hypothesis and the evolution of living systems (10–16). These droplets, having a size in the range ∼1 µm to 30 µm, are charged and are expected to be highly polarizable because of a diffuse counter ion cloud associated with the highly charged polyelectrolyte chains in the polymer complexes (17, 18). It is often said that such droplets are membraneless in the sense that there is no lipid membrane as in vesicles and living cells, but this term does not imply that there is no interfacial layer around the coacervate droplets. We note that high polarizability is an inherent property of many biological materials, and Fröhlich (19) has emphasized the critical importance of this property for basic biological processes.

**Fig. 1.**
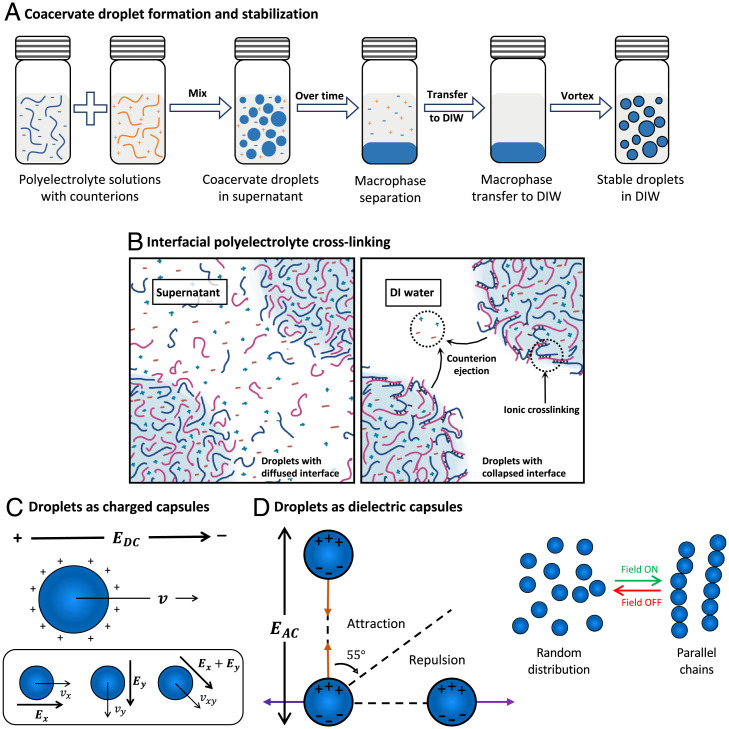
(*A*) Schematic of the stabilization technique; coacervate droplets are formed by mixing two oppositely charged polymers. The droplets coarsen to form a macrophase-separated material. The coacervate macrophase is collected, transferred to DI water (DIW), and vortexed to form stable droplets. (*B*) Illustration of the hypothetical diffuse interface of a coacervate droplet in equilibrium supernatant (*Left*) and proposed hypothesis of its collapse upon transfer to ion-free water due to interfacial ion ejection assisted ionic crosslinking of interfacial chains. (*C*) Illustration of the external electric field-driven coacervate capsules; a positively charged stable coacervate droplet moves along the field lines, and its velocity and direction of movement can be manipulated by manipulating the strength and vector of the applied field. (*D*) Illustration of dipolar interactions between coacervate capsules in external electric field; (*Left*) droplets attract (orange arrows) or repel (purple arrows) each other depending on their positions with respect to the field direction. The transition between attraction and repulsion happens at a critical angle of 55°. (*Right*) dipolar attractions lead to the formation of pearl-like droplet chains upon turning on the electric field.

Although this type of water-in-water emulsion is highly biocompatible and can partition and transport various (bio)chemicals in it (20), this well-known type of droplet structure has an unfortunate attribute that one might think would completely preclude its use as an ER fluid. The surface tension of these particles is remarkably low (14, 15, 21), and unlike vesicles, the droplet interface is expected to be diffuse (22). The droplets readily coalesce upon contact and are unstable to large-scale phase separation. Clearly, some further action must be taken to “stabilize” the droplets to inhibit this coalescence process, at least kinetically. Our approach to this basic problem for coacervate droplets is an important aspect of the present work. We found that we could greatly alter the interfacial properties of the coacervate droplets by simply transferring them to deionized water, a process that renders the interfacial region of the coacervates highly viscoelastic without the need for additives or a chemical reaction to achieve this effect (Fig. 1*A*). Although sometimes resulting in precipitate formation for strong polyelectrolyte coacervates, this simple procedure renders the coacervate droplets formed by weak polyelectrolyte(s) highly stable against coalescence. We hypothesize that this effect arises from the extraction of a significant fraction of the counter ions localized about the droplet interface (in the form of extrinsic ion pairs between the charged polymers and the counterions), which can lead to the formation of physical crosslinks between the polyelectrolytes of opposite charge (in the form of intrinsic ion pairs) (Fig. 1*B*) (23–25). This cross-linked layer appears “self-sealing” in the sense that it prevents additional extrinsic ions within the coacervate droplet from migrating out into the deionized (DI) water. Importantly, the coacervate droplets remain electrostatically charged and polarizable, and we find that they can be readily manipulated individually (Fig. 1*C*) and organized collectively (Fig. 1*D*) with low external electric fields on the order of 10 V/cm.

**Fig. 2.**
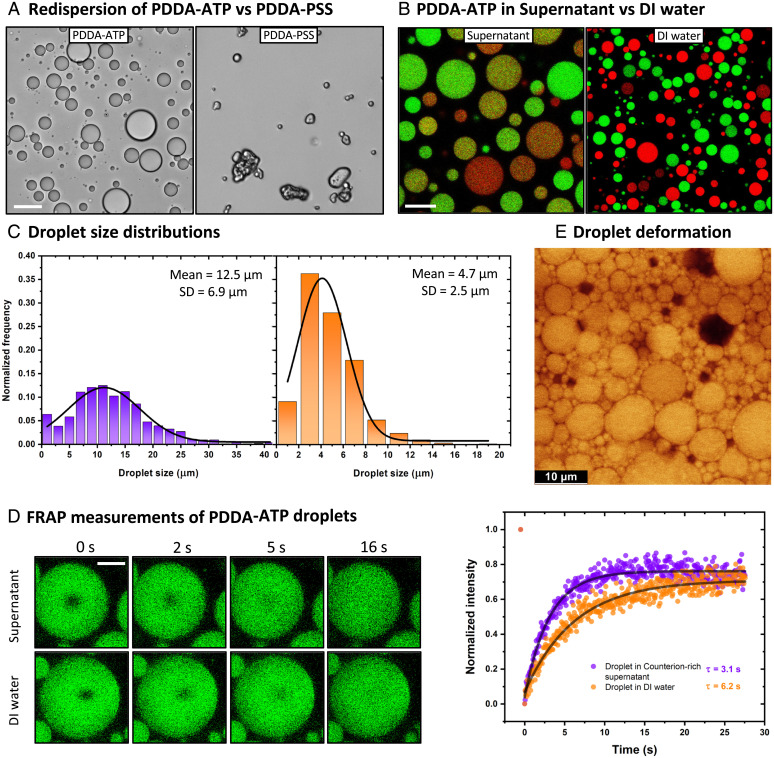
(*A*) Bright-field micrographs of different coacervate systems upon macrophase transfer to DI water where stable droplets are formed by PDDA-ATP (*Left*), while solid precipitates are formed by PDDA-PSS (*Right*). Scale bar: 20 µm (*B*) Confocal micrographs showing coalesced bicolored droplets in the supernatant (*Left*), which are absent in DI water (*Right*) owing to interfacial stabilization. Scale bar: 20 µm (*C*) Droplet size distribution of PDDA-ATP droplets in counterion-rich supernatant (*Left*) and stable resuspension in DI water. SD, standard deviation. (*D*) Time series of confocal micrographs taken right after photobleaching a small area in coacervate droplets prepared in the supernatant (*Top*) and stabilized in DI water (*Bottom*). The fluorescence recovery in the photobleached areas is plotted on the *Right* showing similar recovery time scales. Scale bar: 20 µm. (*E*) Confocal micrograph showing deformed stabilized droplets after centrifugation inside a thin glass capillary.

As summarized in *SI Appendix*, Table S1, we started screening coacervates formed by polyelectrolytes of different strengths and chain lengths. First, aqueous solutions of polyelectrolytes of opposite charges were mixed, leading to liquid–liquid phase separation and formation of spherical coacervate droplets (polymer enriched phase) suspended in counterion-rich supernatant (polymer depleted phase). In some cases, irregularly shaped gels or precipitates were formed, and the addition of salt was required to form spherical droplets instead (*SI Appendix*, Figs. S1 and S2). Dispersed in the supernatant, the coacervate droplets were prone to coalescence even without any external field, and akin to oil droplets in water, they coalesced within minutes to form larger droplets (Video S1). The dispersion ultimately macrophase separated into two layers within hours (*SI Appendix*, Fig. S3*A*). The macrophase separation can be accelerated by centrifuging the suspension. We transferred this coacervate macrophase to DI water and, upon vortexing, obtained droplets that were resistant to coalescence (Fig. 2*A* and *SI Appendix*, Fig. S4). Not all the polyelectrolyte pairs chosen formed stable droplets, however. Some of them, usually those with strong ionization or longer chain, ended up forming precipitates (Fig. 2*A* and *SI Appendix*, Fig. S4). This difference in the behavior of such salty coacervate systems helped us identify the source of droplet stabilization caused by immersion of the droplets in DI water.

We chose to further explore a previously well-studied coacervate system formed by mixing poly (diallyldimethylammonium chloride) (PDDA) and adenosine triphosphate (ATP) that has been shown to share similar physicochemical characteristics with intracellular compartments (16). We obtained stable spherical droplets from this system upon resuspension of coacervate macrophase in DI water (Fig. 2*A*) that remained stable even after a month when left under standard laboratory conditions (*SI Appendix*, Fig. S5). We visualize these droplets under a fluorescence microscope by taking advantage of selective partitioning of dye-labeled protein molecules in PDDA-ATP coacervates during phase separation, creating two fluorescently distinct, but otherwise identical droplet populations (*Methods*, *SI Appendix*, Fig. S6). Mixing the two droplet populations in their counterion-rich supernatant resulted in coalescence and the formation of droplets containing both fluorophores (Fig. 2*B*). When we instead mixed two stable emulsions created by macrophase transfer of individual fluorescently labeled coacervate phases in DI water, the droplets maintained their identities for the period of our measurements (timescales on the order of a month), as confirmed by the fact that we did not find droplets containing both fluorophores, even upon vigorous mixing (Fig. 2*B*). Upon centrifuging these droplets at speeds that would otherwise cause macrophase separation of unstable droplets in 5 min, we found negligible coalescence even after a 30-min run (*SI Appendix* for details, *SI Appendix*, Figs. S3*B* and S7), demonstrating that there is a considerable barrier to droplet coalescence. We re-emphasize that the droplets in DI water are not forming gel-like particles. The water content of the coacervates decreased only by 5% upon stabilization (*SI Appendix*, Fig. S8), which is not enough to cause their gelation. To further support our inference that the interior of the coacervate droplets remains liquid-like rather than gel-like after immersion into DI water, we used FRAP (standing for fluorescence recovery after photobleaching) (*SI Appendix* for details) to determine and compare the timescale of diffusion of fluorescently labeled polymer chains inside different droplets (Fig. 2*C*). Our FRAP observations indicate that the diffusion coefficient of the polymer in the stabilized droplet ($\tau^{\mathrm{DIW}}=6.20\;s$ and $D^{\mathrm{DIW}}=0.16\;\mu m^{2}/s$) is on the same order of magnitude as that inside an unstable droplet suspended in the counterion-rich supernatant ($\tau^{\sup}=3.09\;s$ and $D_{\mathrm{FRAP}}^{\sup}=0.32\;\mu m^{2}/s$) and remain similar even after 18 days of incubation at standard laboratory conditions (*SI Appendix*, Fig. S9). These observations are also consistent with bulk rheological measurements (experimental details in *SI Appendix*, Fig. S10), as well as with our experiments with droplet shape deformation and reformation, which we discuss later in the text. More convincingly, we incorporated fluorescent particles into the droplets, which allowed us to directly observe electric field-induced fluid convection within the droplets. We describe these experiments later below in connection with the description of other electric field effects on the coacervate droplets. Thus, the droplets remain liquid-like after resuspension in DI water, but with enhanced interfacial viscoelastic properties.

What interfacial properties would inhibit droplet coalescence? Emulsions are usually stabilized with electrostatic or steric repulsion between the droplets. Theoretically, we might expect the stability to increase with surface charge (26). For our PDDA-ATP droplets, we find the magnitude of the apparent zeta potential decreases upon introduction into DI water, which suggests that droplet charge is not responsible for their stabilization and some other forces are involved in inhibiting coalescence (*SI Appendix*, Fig. S11). We further hypothesize that on redispersion of coacervates in DI water, the counter ions localized to the droplet interface in large measure transfer to the bulk ion-free (DI) water. This loss of interfacial counter ions would allow direct associations between the ionic monomers of the positively and negatively charged polymer chains become energetically favorable (so-called intrinsic ion association) (23–25), possibly creating a physically cross-linked layer and thus providing a strong steric repulsion between the droplets (Fig. 1*B*) associated with droplet interfacial viscoelasticity. We emphasize that the stabilized droplets are liquid-like inside. Moreover, they appear to remain in such a state for years as found in our 21-mo-old sample, where the spherical shape of these droplets was preserved (*SI Appendix*, Fig. S12). Since we do not have a direct visualization of the relative salt ion concentration at this point, we can only guess that enough salt ions remain inside the stabilized droplet to keep the interior liquid-like. Further measurements are required to test and understand this hypothesis of interfacial cross-linking and the origin and degree of the salt partitioning within the coacervate droplets.

There is an interesting and useful consequence of this proposed physically crosslinked interface—the interface must gain some viscoelasticity due to network formation. This might help in explaining how the droplets avoid coalescence even under externally applied pressure. To visualize the effect of interfacial elasticity, we filled a thin glass capillary with coacervate droplets encapsulated with the fluorescently labeled proteins, applied external pressure on it using high-speed centrifugation, and observed it under a confocal microscope. After 20 s of spinning at 2,500 × g, we found at the bottom of the capillary highly deformed droplets that remained stable in their nonspherical shapes with occasional coalescing throughout the imaging process (∼30 min) (Fig. 2*E*). We emphasize that this deformation is not permanent—when the capillary was broken so that the droplets can redisperse in bulk solution, they rapidly recover their spherical shape. Tellingly, when the droplets settle by gravity and then come into contact with the cover glass, they remain nearly spherical, so their interfacial stiffness must be appreciable to prevent any distortion in their shape as droplets normally do under gravity on surfaces with partial wetting conditions (*SI Appendix*, Fig. S13). Thus, the droplet distortion seen in Fig. 2*E* is a confinement effect associated with strong interdroplet interaction when their concentration is significantly high. This behavior is characteristic of droplets having a viscoelastic interface, such as Pickering emulsions and foams and phase-separating polymer blends undergoing transesterification where a similar viscoelastic layer arises that impedes particle coalescence (27–30). Since these inferences of the fluid-like nature of the droplet interior are indirect, we performed measurements to unequivocally demonstrate the fluidity of the interior of the droplets using an electric field in the later part of this article.

**Fig. 3.**
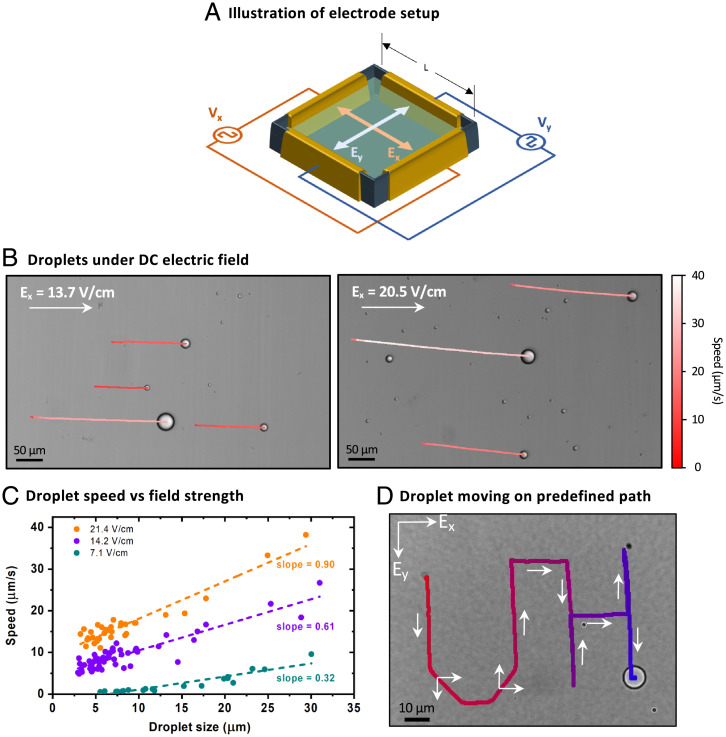
(*A*) Illustration of the setup used to apply electric field on coacervate droplets sedimented at the bottom of the well. Here, an orthogonal field can be applied via two independent pairs of electrodes, and the x and y position of the droplet can be controlled independently. (*B*) Bright-field image of stable coacervate droplets moving in straight lines parallel to the external electric field of strengths. The images show trajectories of individual droplets over a period of 10 s. The trajectories are color-coded with their instantaneous speed. Note that the larger droplets move faster than the smaller ones and cover longer distances. (*C*) The average speed of droplets plotted against their size at different voltages. The dotted line is a linear fit to the data points, and the slope of this fitting line is mentioned. Note that the magnitude of slope increases proportionally with the applied voltage. (*D*) A stabilized droplet of 10-µm diameter moving on a predefined path writing characters 'UH' using a programmable microcontroller and circuit encoded with information to turn on and off $E_{x}$ and $E_{y}$ at different times, as shown using white arrows. $\left|E_{x}\right|=\left|E_{y}\right|=13.7\;\text{V}/\text{cm}$.

In this section, we show a key consequence of droplet stabilization. When we subjected the stabilized droplets to direct (DC) and alternating (AC) electric fields, we could control their position and organization in solution. An aqueous suspension of stabilized droplets was pipetted into a square well in a 96-well plate with copper foil tape on the four walls, and each pair of opposite walls were connected to independent voltage sources (Fig. 3*A*, *Methods*). In the absence of an electric field, the sedimented (but not adsorbed) droplets on the cover glass were found jiggling randomly. As soon as the DC electric field was turned on, the droplets started to propagate along nearly straight trajectories in the direction of the applied electric field vector. As expected from a charged particle in an electric field, the droplets moved with a field strength-dependent average velocity (Figs. 1*C* and 3*B*). Balancing drag forces at a low Reynolds number in the equation of motion gives a linear relationship between droplet velocity and its diameter with a slope directly proportional to the applied field strength (*SI Appendix* for details). We found that the coacervate droplets followed this relation very well (Fig. 3*C*). The magnitude of the droplet velocity was found to vary linearly with the droplet size—larger droplets moved faster than the smaller ones, even when in close vicinity of each other (Video S2). We are therefore looking at the response of individual droplets and not just the convective motion of the underlying fluid, i.e., electro-osmotic flow.

**Fig. 4.**
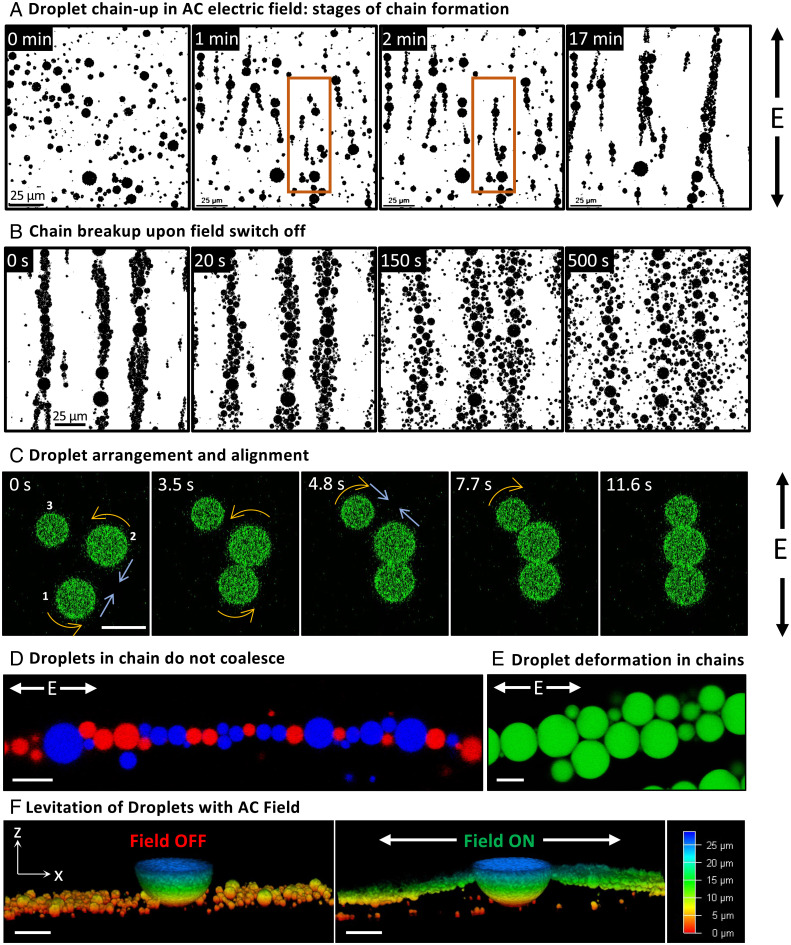
(*A* and *B*) Time-lapse confocal micrographs showing (*A*) chain formation by droplets upon turning on the AC electric field (35.7 V/cm at 100 kHz) and (*B*) chain break up upon turning off the field. Images were binarized using NIH ImageJ for clarity. During chain formation in *A*, in the first transition state from 0 min to 1 min, oligomers are formed by neighboring droplets. In the second transition state from 1 min to 2 min (and beyond), the oligomers join (see orange box) end-to-end with others leading to chain elongation. Finally, at long times (shown at 17 min here), the chains aggregate by diffusing normal to the dipole vector to form bundles. In *B*, the smaller droplets diffuse apart faster compared to the larger ones due to their relatively higher Brownian diffusion. (*C*) Dipolar interaction induced the motion of three neighboring droplets leading to the formation of a chain under an alternating electric field of strength 35.7 V/cm at 100 kHz. The blue arrows indicate the direction of motion of droplets due to attractive interactions and the yellow arrows indicate the rotation of chains to align them (i.e., the chain’s overall dipole moment) parallel to the field lines. At t=0, there is an attractive interaction between droplet pairs 1 and 2 and 1 and 3, while a repulsive interaction exists between the pair 2 and 3. Scale bar: 5 µm. (*D*) Merged confocal image from two different channels showing a chain of droplets containing BSA-CF488A and BSA-CF640R (false-colored red and blue, respectively) under field strength of 17.9 V/cm at 100 kHz. Note that no droplet contains both the fluorophores. Scale bar: 10 µm. (*E*) Confocal image showing interfacial deformation of BSA-CF488A labeled stable droplets under field strength of 35.7 V/cm at 100 kHz. It was observed that a higher size mismatch between the neighboring droplets caused larger deformations. Scale bar: 10 µm. (*F*) Three-dimensional confocal images showing the levitation of smaller droplets to the equator of a giant droplet of diameter ∼60 µm upon turning on the electric field (44.6 V/cm at 100 kHz). The field is along the x axis, and the height of the structure is color-coded as shown on the scale. Lateral scale bar: 30 µm.

Next, using two independent orthogonal electric fields, namely, E_x_ and E_y_, driven by a programmable microcontroller and relay switches, we were able to control the motion of the droplets on a two-dimensional plane that allowed us to steer them on predefined paths (Fig. 3*D* and S3 and S4). We envision that these stabilized droplets, encapsulated with functional biomolecules such as enzymes, can be driven around obstacles and through mazes to transport materials. In some cases, we observed a slight but random deviation from the predefined path, which we attribute to the competing Brownian motion of the droplets and convective flows developed in the solution. However, we did not observe breakup or wetting of any droplet in all these experiments. To assemble stabilized coacervate droplets in chains in a way similar to dielectric particles in ER fluids, we applied an alternating electric field of a square waveform. In such a condition, a polyelectrolyte coacervate droplet can be considered a dielectric particle in water whose dipole vector is aligned parallel to the applied field vector, leading to dipolar attractive and repulsive interactions among the droplets depending on their relative positions (Fig. 1*D*, *SI Appendix* for details) (1). These interactions at a nominal field strength of ∼20 V/cm, along with assistance from Brownian motion, allow the formation of droplet chains. At frequencies much higher than the single-droplet translation response (>100 Hz), we observed distinct transient stages of chain formation: elongation and bundling (Fig. 4*A* and Video S5). When the field is turned off, the droplets disassemble from the chains and go from their highly anisotropic state back to the near-isotropic distribution after an extended time that depends on solution conditions (Fig. 4*B* and Video S6). The suspension self-heals, in this case, to its unstructured state when the electric field is turned off.

To better visualize this aggregation behavior, we collected a time-lapse video of three neighboring droplets aligning under an external field of strength of 17.9 V/cm at 100 kHz (Fig. 4*C* and Video S7). Here, we could clearly see that the interaction between the droplets was position-dependent: droplets in the pair 1 and 2 attracted each other as they made an angle less than 55° (critical angle) with field lines and formed a chain, while the droplets in the pair 2 and 3 repelled each other initially as the angle they formed with the field lines was more than 55°. Only upon aligning at an angle close to the critical angle the droplet #3 got attracted to the cluster and joined it (*SI Appendix*, Fig. S14). Moreover, the droplets kept repositioning inside the cluster until the whole chain was parallel to the field lines. Taking advantage of the fact that the droplets were electrostatically positively charged, we observed a worm-like motion of the chains under the AC field with a DC bias (Video S8). The AC field kept the chains intact, while the DC field allowed us to move them along the DC bias field lines collectively. We note that the particles do not fuse permanently, unlike other field-structured particle systems. Instead, the viscoelastic interface of the droplets allows them to deform while avoiding coalescence (Fig. 4 *D* and *E*). At a high field strength of 71.4 V/cm, some of the big droplets, however, coalesced to form giant yet nonwetting droplets of 50 µm to 150 µm in diameter, and in their wake, a slew of smaller droplets moved to the poles of these giant droplets giving rise to levitated chains (Fig. 4*F* and Video S9).

**Fig. 5.**
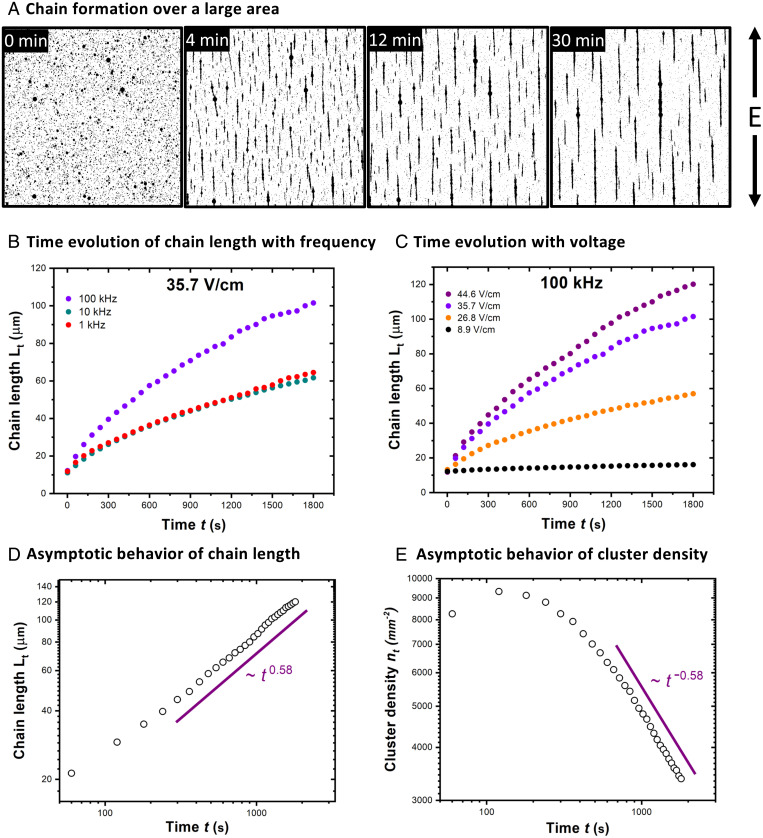
(*A*) Time-lapse images of chain formation by stabilized coacervate droplets at 44.6 V/cm at 100 kHz (ϕ ∼ 0.07) in a large area showing long chains aligned parallel to the applied field. The images (550 µm × 550 µm) are cropped from their original version (4.2 mm × 3.7 mm). (*B* and *C*) Average chain length as a function of time plotted for different frequencies (1 to 100 kHz) at constant voltage (35.7 V/cm) (*B*) and different voltages (8.9 to 44.6 V/cm) at a constant frequency (100 kHz). (*D* and *E*) Average chain length (L_t_) and cluster density (n_t_) at 44.6 V/cm at 100 kHz as a function of time from *A* plotted on a log-log scale. They closely follow an asymptotic behavior as predicted by Miguel and Pastor-Satorras (34) for anisotropic chain diffusion model.

We found that the process of chain formation is diffusion-limited. When the field was briefly switched off so that the chains began disintegrating and the field switched back on again, we found that the chain growth was significantly accelerated compared to the case in which the droplets were initially randomly distributed. A previous observation of ER fluids has shown that the chain growth kinetics of colloidal dielectric particles generally depends strongly on both the amplitude and the frequency of the applied field (2, 5), and we likewise find this to be true for our counter-ion-stripped coacervate droplets. We sampled sedimented droplets within the limit of low droplet concentration with an effective area coverage ϕ of ∼0.04 to 0.08. At 44.6 V/cm and 100 kHz, chain formation was observed within seconds after turning on the electric field; droplets started clustering into chains (Fig. 5*A* and *SI Appendix*, Fig. S15 and Video S10). The chains grow longer with time by joining end-to-end with other chains. The droplet chains were relatively flexible in nature, and we often found that they undulated due to thermal fluctuations (*SI Appendix*, Fig. S16 and Video S11). These shape fluctuations can be attributed to Landau–Peierls instabilities that arise in one-dimensional structures (31, 32). The fluctuations diminished in thicker chains (Video S12). This is expected from fibrous materials, where the stiffness of the self-assembled structure increases with increased bundling (33).

Next, we consider how similar or different the field-directed self-assembly process of the coacervate droplets to the self-assembly of solid particles. To do so, we analyze chain formation from the perspective of particle-like phase separation where the average chain length, L(t) [classically termed as cluster size (34), *SI Appendix* for details], was calculated as a function of time, t, and found that it varies with the frequency and the voltage of the applied field. At a field strength of 35.7 V/cm, the chain growth increased upon increasing the frequency from 10 kHz to 100 kHz (Fig. 5*B*). This might seem counterintuitive, as the polarizability (β) usually decreases at high frequency (18). However, the diffusion of ions has been argued to lead to an electrohydrodynamic distortion of the electric layer (modeled as a double layer) of counter ions around the droplet poles, which is predicted to decrease the strength of the attractive dipolar interaction between the polarizable droplets (*SI Appendix*) (2, 5). This effect will lead to an enhancement in the rate of growth of the droplet chains at frequencies higher than the ion-diffusion timescale, and an optimum frequency can be expected for the fastest growth of the chains by balancing different driving forces (*SI Appendix* for details). Correspondingly, by holding the frequency constant at 100 kHz, we varied field strength from 8.9 V/cm to 44.6 V/cm and found that the chains grew faster at high field strengths (Fig. 5*C*). This faster growth is expected as a higher field strength would induce larger dipoles leading to a greater attraction between the droplets. The average length of the droplet chains, L(t), increases at long times as $t^{\lambda }$, with λ=0.58±0.04 (Fig. 5*D*). This coarsening exponent is strikingly similar in magnitude to the estimate of 0.61 found by Miguel et al. (34) for anisotropic diffusion of rodlike particles while clustering under an electric field due to hydrodynamic interactions. We also estimated the number density of chains per unit area, n(t), as a function of time t, and found it to scale as, n(t) ∼$\;t^{-0.58}$ (Fig. 5*E*). Note that the predicted cluster size exponent λ from the cluster–cluster aggregation model by Vicsek et al. (35) has the same magnitude as the density exponent, consistent with our observations. It is worth noting that the chain formation appears to require a critical field strength that depends on the particle size (*SI Appendix*, Fig. S17). The particle size and the associated polydispersity in the sample can manifest themselves in the rate of chain formation and seems to affect the rate in our experiments at low field strengths (*SI Appendix*, Fig. S18).

Given the high propensity for chain formation by these droplets at relatively low field strengths, we conclude that these droplets are highly polarizable (*SI Appendix*). Moreover, we found the emergence of convective motion of tracer particles in the interior of the coacervate droplets when kept under the uniform E-field, which we attribute to electrohydrodynamic flows (EHD; *SI Appendix* for details, Video S13) (36, 37). It seems likely that the convective motion within (and around) the droplets also contributes to the field-induced translation of the coacervate droplets, so there are nontrivial EHD aspects of understanding the effective charge of these complex droplets (*SI Appendix*). Thus, our emphasis on droplet charge in explaining the droplet drift is clearly overly simplistic, and further quantification of the droplet polarizability, and its physical origin, is required to fully disentangle the charge-induced versus the dielectrophoretic contributions to the drift velocity. The quantification of these contributions to field-induced force must await future study. Finally, we would like to note that the convective flow inside the droplets serves as another direct proof of the liquid-like core of these stabilized droplets in addition to the FRAP measurements. Since the tracer particles move even in the close vicinity of the droplet interface, we hypothesize that the proposed interfacial crosslinking upon DI water transfer is limited to a few molecular length scales. Moreover, when small uncharged and charged molecules were introduced into the droplet suspension after stabilization, they diffused and partitioned into the droplets, indicating a minimal diffusion barrier, thus further strengthening this hypothesis of a very thin crosslinked interface (*SI Appendix*, Fig. S19). Future studies using neutrons and X-ray scattering may elucidate the physical length scale of this crosslinking and its change with time, if any.

We have demonstrated the unique properties of coacervate droplets when “stabilized” through immersion into water, pressumably stripping off some of their counter ions from the interfacial region of the droplets to form physical crosslinks in this region, provides an approach for creating stable “membraneless” compartments for conveying a wide range of cargos for a myriad of potential applications. The proposed mechanism of droplet stabilization is not entirely understood, and our description is based on a working model, indirectly supported by many observations described in the present paper. The stabilization mechanism appears to be quite general as it works for a wide range of polyelectrolytes, arresting them in a nonequilibrium state. Although this out-of-equilibrium system may appear unique, it is quite analogous in some ways to polyelectrolyte multilayer films formed by the layer-by-layer sequential deposition of polyelectrolytes having opposite charges. The charged polymers in these layers interpenetrate strongly and account for the high stability of these layers. In particular, we anticipate the hypothetical gel-like layer of the DI water stabilized coacervate droplets analogous to charged polymers that are intermixed at the molecular length scales (38–40). Preparation of these films involves washing in DI water between each deposition step “to remove excess polyelectrolyte from the just deposited layer,” and this washing step has been found to stabilize these films (41, 42). Although the precise structure of the interfacial layer is unknown, this stabilization is consistent with our hypothesis that exposing the complexes to relative pure water has the effect of depleting polyelectrolyte–counter ion complexes and replacing these with direct ionic associations between the polyelectrolytes.

The minimalistic coacervate droplet stabilization method that we discussed could have ramifications for the spontaneous formation of semipermeable interfaces in early protocells before the evolution of complicated structures like lipids. Previous attempts at droplet stabilization, for example, using liposomes, amphiphilic polymers, and complex microfluidic techniques, have shown excellent partitioning of small molecules, but these alternative stabilization methodologies achieved “stability” apparently for relatively short times and under quiescent mixing conditions (43–45). The viscoelastic behavior of our hypothetical ionically crosslinked interfaces, along with a permanent surface charge and high polarizability, enable the manipulation of individual and collections of droplets using an externally applied electric field. Previous attempts to control coacervate droplets with an electric field simply resulted in droplet elongation, coalescence, or rupture, and these effects were often irreversible in nature (46, 47). The field-structured coacervate chains of droplets are remarkably persistent in time, and we did not see any droplet coalescence in the chains over a wide range of applied field strengths. This robustness of these field-induced droplet assemblies was confirmed by fluorescence imaging of mixed-labeled droplets and their interfacial deformation. We stress that it is highly advantageous for applications that the required field strength for this kind of ER fluid is two orders of magnitude smaller than in previous studies of colloidal particles, where field strengths on the order of 10 kV/cm were typically utilized (2–5). We attribute this capability of coacervates to the high dielectric constant of polyelectrolyte materials (*SI Appendix*). Field strengths on the order of 10 V/cm or less make these materials much more suitable for biocompatible (personal care and medical) applications. Chain formation under an electric field has been found in biological systems, such as the suspension of cells in a buffer (48–50), so the droplet system that we study promises to be a model system for many naturally occurring biological structures and synthetic protocells in the form of phase-separated coacervate complexes (51–54).

We finally note that Fröhlich’s (19) seminal modeling of large-scale collective phenomena in living systems as arising from electric-field-induced changes in polarization state is becoming more appreciated scientifically (55–59), and this motivates the study of whether the coacervate materials can exhibit the electric-field-induced switching in the polarization state that he and others following him have suggested underlies many important biological processes.

## Methods

### Coacervate Droplet Formation by Phase Separation and Droplet Stabilization.

Coacervate droplets in supernatant for different polyelectrolyte combinations were prepared by adding polyanion to DI water, followed by the addition of polycation. Unless stated otherwise, the final concentration of polycation and polyanion added was 20 mM on a charge basis (equivalent to 20 mM PDDA on a monomer basis and 5 mM ATP). For PDDA-ATP coacervates, NaOH was added to the ATP solution at a concentration of 10 mM before the addition of PDDA. A 1 mL of total polyelectrolyte solution yielded around 11 μL of PDDA-ATP coacervate macrophase. For PDDA-sodium poly (styrene sulfonate) (PSS) coacervates, KBr was added to the stock solutions of polyelectrolytes at a concentration of 0.5 M to 4 M before mixing them to form coacervates (or precipitates). All samples were made in plastic Eppendorf centrifuge tubes, and the solutions were vortexed after each component was added to ensure proper mixing. In all the cases, turbidity in the solution appeared right after the addition of polycation and was evidence of coacervate (microphase) formation. To form the macrophase, the suspension was centrifuged at 1,000 × g for 5 min, and the macrophase, being denser than supernatant, was collected at the bottom of the tube. To make stable droplets, the required amount of the coacervate macrophase was pipetted out and added to a vial containing DI water that was then vortexed to allow the breakup of the macrophase into droplets.

### Droplet Coalescence Experiments with Fluorescently Labeled Coacervate Droplets.

Experiments to check for droplet coalescence under mixing were done with dye-labeled bovine serum albumin (BSA) molecules. A 1-mg/mL solution of BSA-CF488A or BSA-CF640R was added at a final concentration of 5 nM to the precursor solution (polyanion/salt/base solution), and polycation was added at last. Given the high level of the partitioning of BSA in coacervates, the final concentration of BSA in the coacervate phase was estimated to be around 0.5 μM. For FRAP measurements, FITC-labeled CMDex150 was added to the precursor solution prior to polycation addition to a final concentration of ∼0.1 mM (monomer basis) in coacervate macrophase. This quantity was very low compared to the final PDDA and ATP concentration in coacervate macrophase (∼2 M monomer basis) to not cause any major phase change, but still large enough for imaging.

### Microscopy.

Coacervate droplets were imaged on cover glass bottom, black-walled plates (Cellvis P96-1.5H-N). The wells were washed in sequence with NaOH (1 M), HCl (1 M), DI water, and 2% (mass/volume) polyvinylpyrrolidone (PVP) solution in water (for at least 2 h) to minimize droplet wetting on cover glass. Brightfield images were captured on an inverted microscope (DMi8, Leica) fitted with a white light lamp (X-Cite LED, Excelitas) and a camera (K5 sCMOS, Leica). Fluorescence microscopy was done on a laser scanning confocal microscope SP8, Leica) equipped with 488-nm and 640-nm excitation lasers used for imaging BSA-CF488A and BSA-CF640R, respectively, with proper excitation/emission filter sets. LAS X software from Leica Microsystems was used to acquire images/videos, and an oil-immersion objective lens of 63× magnification was used for imaging unless specified otherwise.

### Light Scattering Measurements.

Turbidity and zeta-potential measurements of coacervate droplets were performed on an electrophoretic light scattering instrument (Litesizer 500, Anton Paar) equipped with a 658-nm laser. Turbidity was measured by recording the amount (%) of light transmitted through the sample. Zeta-potential measurements were performed in the proprietary omega-cuvettes.

### Experiments with Electric Fields.

A simple customized setup for applying the electric field on coacervate droplets (as sketched in Fig. 3*A*) was made in-house by gluing adhesive double-sided conductive copper tapes on the four walls of a well in the 96-well plate (#89626, ibidi GmbH) for DC field experiments with well dimensions as 7.4 × 7.4 mm^2^ or on two opposite walls of a chambered coverslip (#81816, ibidi) for AC field experiments with a wall-to-wall distance of 5.7 mm. Each well was pretreated with PVP using the protocol described above prior to taping copper on the walls and was filled with the coacervate droplet suspension. They were then covered with the 3M magic tape to minimize evaporation of the suspended fluid. The free ends of the electrodes (copper tape) were connected to wires that connect to an external circuit. After taping with copper foil, the final distance between the walls was measured as 7.3 mm for 89626 wells and 5.6 mm for 81816 wells. For experiments involving DC electric field, the external circuit was composed of an Arduino microcontroller that controlled relay switches connecting a variable DC voltage source to the copper electrodes. The microcontroller was programmed using Arduino IDE, and the field was created between a pair of electrodes to move the droplet in a particular direction (see Fig. 3*B*). The system was studied under the brightfield microscope with a 20× or 40× air objective. Droplet velocity was calculated by analyzing the distance covered by the droplets after turning on the field. For experiments under AC electric fields, the external circuit consisted of a function generator (Rigol DG4162), where a square waveform of different amplitudes and frequencies was used. A high-speed bipolar voltage amplifier (A-301HS, A. A. Lab Systems Ltd.) was used to amplify the output voltage of the function generator at a given frequency. The final maximum frequency and a voltage output that the amplifier could generate were 200 kHz and 200 V (= 400 V peak-to-peak). The images were acquired using automated stage movement and sequential scanning under the confocal microscope with a 10× dry objective lens to observe chain formation over a large area. The obtained images were stitched and analyzed using ImageJ, briefly described in *SI Appendix*.

### Materials.

A drief detail of materials used is as follows: PDDA (molecular mass 8,500 Da, Polysciences, Inc.); PSS (70 kDa, Sigma-Aldrich, Inc.); ATP, PVP (360 kDa) and KBr (TCI America); carboxymethyl dextran sodium salt (CMDex20, 20 kDa, and CMDex150, 150kDa) (tdb Labs); poly epsilon L-lysine hydrochloride (3,500 to 4,500 Da) and hyaluronic acid sodium salt (HA15, 8 to 15 kDa, and HA80, 70 to 80 kDa) (Carbosynth); dye conjugated BSA (BSA-CF488A and BSA-CF640R) (Biotium, Inc.); and DI water (18 MΩ cm, Thermo Scientific).

### Disclaimer.

Certain commercial equipment, instruments, or materials are identified in this paper to foster understanding. Such identification does not imply recommendation or endorsement by the National Institute of Standards and Technology, nor does it imply that the materials or equipment identified are necessarily the best available for the purpose.

## Supplementary Material

Supplementary File

Supplementary File

Supplementary File

Supplementary File

Supplementary File

Supplementary File

Supplementary File

Supplementary File

Supplementary File

Supplementary File

## Data Availability

All study data are included in the article and/or supporting information.
