# Supplementary material for: Manipulation of coacervate droplets with an electric field
Source: Proc Natl Acad Sci U S A. 2022 Aug 4;119(32):e2203483119. doi: 10.1073/pnas.2203483119 (PMC9372540; doi:10.1073/pnas.2203483119)
Supplement: Supplementary File [file pnas.2203483119.sapp.pdf]

Supplementary Information:

Manipulation of Coacervate Droplets with an Electric Field

Aman Agrawal[1,*], Jack F. Douglas[2,*], Matthew Tirrell[3], and Alamgir Karim[1,*]

[1]William A. Brookshire Department of Chemical and Biomolecular Engineering, University of Houston, Houston, TX 77204;

[2]Materials Science and Engineering Division, National Institute of Standards and Technology, Gaithersburg, MD 20899; and

[3]Pritzker School of Molecular Engineering, University of Chicago, Chicago, IL 60637

*Corresponding authors:
Aman Agrawal (aagraw27@central.uh.edu)
Jack F. Douglas (jack.douglas@nist.gov)
Alamgir Karim (akarim3@central.uh.edu)

This PDF file includes:

Appendix
Table S1
Figures S1 to S20
Legends for Videos S1 to S13
SI References

Other supplementary materials for this manuscript include the following:

Videos S1 to S13

Appendix

Quantifying Droplet Coalescence under External Pressure

To identify the extent of coalescence at different centrifugal speeds, we used co-localization analysis to quantify the overlapping fluorescence signal from two distinct droplet populations. We used Mander's test, a type of colocalization analysis widely used in biological sciences, where fractional overlap or colocalization of one fluorescent probe with another is calculated. These fractions, called Mander's coefficients, estimate the cellular or subcellular localization of two or more molecules of interest. The notation of Mander's coefficient is M_{12} (and M_{21}) and denotes the overlap of fluorophore one onto fluorophore two (and vice versa). Although in usual situations, these coefficients are not equal, however for systems with symmetric components, like the one we have where the two droplet populations are identical except for their fluorescence signal, these coefficients should be equal.

For our experiments, we vortex mixed the two distinct fluorescently active stable droplet populations of PDDA-ATP (poly (diallyldimethylammonium chloride) - adenosine triphosphate) coacervate (labeled with fluorescently tagged proteins BSA-CF488A and BSA-CF640R, see materials and methods sections for details) in a centrifuge tube (see mixing scheme in **Fig. S6**) and centrifuged the mixture at different speeds for different times. After that, we resuspended the sedimented droplets by gentle vortexing and pipette out the suspension in imaging wells. We used epifluorescence microscopy to image the droplets and acquire 16-bit images from respective filter sets of CF488A and CF640R dyes.

For colocalization analysis, we used the ImageJ plugin JACoP (Just Another Colocalization Plugin).[1] Here, the threshold for each image was manually adjusted and was quite near to Costes' threshold value. Typically, 200 to 500 or more droplets were present in an image. We calculated the M1 and M2 coefficients (i.e., M_{12} and M_{21}) and plotted them in **Fig. S7**.

Fluorescence Recovery after Photobleaching (FRAP)

FRAP measurements provide information regarding fluorescently labeled analytes' diffusivity in a viscous medium. Here a small area (often of circular shape) of the fluorescing region is photobleached in a very short time using high laser intensity. The working principle is based on the rate of recovery of the fluorescence intensity of the photobleached area, as the rate of recovery will depend on the diffusion of fluorescing and photobleached molecules in and out of that area. The faster the recovery, the higher the diffusion coefficient. Various models have been detailed in the literature to extract diffusion coefficients from FRAP data. We used dye-labeled Carboxymethyl dextran (FITC-CMDex150) in our experiments, which partitions preferentially in the PDDA-ATP coacervate and looked at its diffusivity inside the droplets. We chose droplets of diameter around 30 µm and bleached an area with a diameter of 4 µm near the droplet's core. Here, we were not interested in knowing the exact diffusion coefficient; instead, we wanted to see how it changes as we subject the droplets to the process of immersion of droplets in deionized (DI) water to stabilize them.

We adapted a method outlined by McCall et al. with some minor changes.[2] We started by taking pre-bleach images of chosen droplet using a 488 nm laser at 1 % intensity on the confocal microscope setup described in the methods section. We then performed the photobleaching using 100 % of laser intensity from three simultaneous sources (405 nm, 488 nm, and 560 nm) to achieve maximum photobleaching in a short time (500 ms). We collected the post-bleach images at intervals of 70 ms. The normalized average intensity from the bleached region was calculated as:

$$I_{spot}^{norm} = \frac{I_{spot}(t) - I_{spot}(t_{pb})}{I_{spot}(t_o) - I_{spot}(t_{pb})} \tag{1}$$

where, $I_{spot}(t)$ is the average fluorescence intensity of the bleached region at time t and $I_{spot}(t_o)$ and $I_{spot}(t_{pb})$ are the average fluorescence intensity of the bleached area in last pre-bleach frame and first post-bleach frame, respectively.

Unwanted photobleaching inevitably occurs during post-bleach imaging and must be accounted for. It was normalized by calculating the photobleaching in a control droplet (usually a neighboring droplet) over the course of the post-bleach image acquisition process as:

$$f_{photobleaching} = \frac{I_{control}(t)}{I_{control}(t_o)} \tag{2}$$

where, $I_{control}(t)$ is the average fluorescence intensity of the control droplet at time t. The photobleach-corrected normalized average fluorescence intensities are then calculated as:

$$I_{spot,PBC}^{norm} = \frac{I_{spot}^{norm}}{f_{photobleaching}} \tag{3}$$

and are plotted against time in **Fig. 2d** for unmodified droplets in the supernatant and ion-stripped stable droplets in DI water. The obtained data points were fitted to an exponential function with a single time constant, τ, as shown by solid curves in **Fig. 2d**. The time constants are calculated and were found to be 3.09 s and 6.20 s for droplets in the supernatant and DI water, respectively. Diffusion coefficient (D_{FRAP}) varies with the time constant of recovery as,

$$D_{FRAP} = \frac{l^2}{4\tau} \quad (4)$$

where l is the radius of the bleached area and the diffusion coefficient is assumed to scale inversely to the solution viscosity,

$$\eta \propto \frac{1}{D}. \quad (5)$$

Thus, the "apparent viscosity" is directly proportional to the recovery time constant. For fresh (unstable) PDDA-ATP droplets in their equilibrium supernatant, $\tau^{sup} = 3.09\, s$ and $D_{FRAP}^{sup} = 0.3\, \mu m^2/s$, while for stable droplets in DI water, $\tau^{DIW} = 6.20\, s$ and $D_{FRAP}^{DIW} = 0.16\, \mu m^2/s$. Thus, for our system, the viscosity of the core of the droplets increases roughly by a factor of 2 upon stabilization in DI water. This increment can be attributed to a partial loss of counter-ions to the otherwise ion-free water. More importantly, this indicates that some counter-ions are still inside the droplet, preventing crosslinking of polyelectrolyte chains and keeping the droplet's core liquid-like. This model is further supported and explained by bulk rheology experiments in the next section. Moreover, when the measurements were carried on the stabilized droplets aged for 18 days, no significant change in the recovery time constant was observed ($\tau^{DIW\ 18\, days} = 7.8\, s$ and $D_{FRAP}^{DIW\ 18\, days} = 0.13\, \mu m^2/s$). Thus, it can be inferred that the counterions likely remain partitioned inside the droplets even for days after "stabilization".

Bulk Rheological Measurements

We wanted to quantify the bulk rheological properties of coacervate droplets before and after stabilization and compare it with our FRAP measurements. Hence, we performed viscometry and oscillatory rheology measurements of coacervate macrophase to determine viscosity and loss and storage moduli of coacervate phases. The measurements were carried out on a rheometer using a cone and plate geometry (radius 20 mm, cone angle 2°) at 25 °C. A solvent trap was used to minimize water evaporation from samples during measurements. Coacervate samples of PDDA-ATP were prepared at 20 mM polymer concentration, and the macrophase was separated from equilibrium supernatant by centrifuging the sample at 3000 x g for 2 min. For macrophase separation of stabilized droplets, samples were centrifuged at 6000 x g for 2 h to force coalescence and macrophase separation of the droplets. The macrophase-separated samples were kept in isolation for 24 h at 25 °C before performing the measurements.

The viscosity was measured at controlled frequency/strain with logarithmically varying shear rates going from 0.2 s^{-1} to 200 s^{-1} while ramping up and 200 s^{-1} to 0.2 s^{-1} while ramping down. The step time (delay) was chosen as 50 s to provide enough time for equilibration. The viscosity values obtained are plotted against shear rate (s^{-1}), as shown in **Fig. S10**. Note that the viscosity values go down initially, for example, in the case of coacervate macrophase from counterion-rich supernatant from ≈ 60 mPa.s at a shear rate of 0.2 s^{-1} to ≈ 40 mPa.s at 1.2 s^{-1} and remained constant thereafter for two decades of frequency. This change in viscosity at low shear rates point towards shear thinning behavior and can be of interest for future research. For the counter-ion stripped stable droplets, the bulk viscosity of the corresponding macrophase is ≈ 120 mPa.s at high shear rates, about three times higher than that of macrophase from the supernatant. Compared to the results from FRAP measurement, where the viscosity only doubles upon stabilization, the higher increment here in macrophase viscosity can be attributed to the mixing of counterion-stripped interfaces with the bulk of droplets upon forced macrophase separation leading to a lower bulk counter-ion density. Nevertheless, the results show that there is no order of magnitude change in viscosity of these droplets upon stabilization which means that they maintain a liquid core similar to the unmodified coacervates.

We then performed oscillatory frequency sweeps at 25 °C to determine the storage (G') and loss (G'') moduli at a strain of 10 %. This value was well within the linear viscoelastic regime determined by amplitude sweeps carried out at ω = 10 rad/s with γ ranging from 1 % to 60 %. **Fig. S10** shows the storage and loss moduli from the two samples plotted against frequency ranging from 0.01 Hz to 10 Hz. Here as well, we do not see any significant change in the structure of coacervate macrophase for stabilized droplets compared to unmodified ones (i.e., from counterion-rich supernatant). These results are important as they show that while some counter-ions escaped the coacervate droplet during stabilization, the desalting happened primarily at the interface and the bulk salt ions are majorly intact in the interior of the coacervate droplet (and thus the macrophase), preventing crosslinking of the polyelectrolyte chains in bulk and preserving the liquid core.

Imaging in Capillaries

Stabilized coacervate droplets fluorescently labeled with BSA-CF640R were filled inside a thin-walled square cross-section borosilicate glass capillary (#8280 and #8290, Vitrocom Inc, USA). The capillaries were kept open on both ends, and the droplet suspension was pipetted in, after which the capillary was sealed on both ends using capillary wax (#CW08, Charles Supper Company, Inc., USA). After that, the capillary was centrifuged by placing it in a plastic centrifuge tube cushioned with cotton to save the capillary from breaking. It was then glued to a glass slide for protection, mounted on the LSCM microscope stage with the oil-immersion objective lens, and imaged at different z-planes to ensure that the wall effects were excluded.

Image Analysis Using Fiji (ImageJ2)

The acquired images were around 4 x 4 mm^2 with a pixel size of about 1.04 µm. Image analysis was performed in Fiji, an open-source image analysis software based on NIH ImageJ. Each image was carefully 'thresholded' to convert to a binary image for further processing. Droplets and chains were identified using the "Analyze Particles" option with a minimum particle area of 30 μm^2 corresponding to a minimum particle radius of 3.1 µm, almost 3 pixels units, and different identifiers, such as *Area* and *Feret,* were used as chain parameters, corresponding to the projected area and chain length, respectively. The sum of the projected area was used to calculate the droplet area coverage, ϕ, and was kept between 0.04 to 0.08. To get droplet trajectory, as shown in **Fig. 4c**, coordinates of the center of each droplet were obtained based on the methods described in the *Analyze Particles section* and the plotted as a function of time, *t*.

Charged Spherical Droplet Subjected to a Uniform External Electric Field

Stabilized coacervate droplets of PDDA-ATP can be considered positively charged particles with the charge distributed uniformly over the surface of the droplets. For such a particle moving under an external field, there are two main forces acting on it, i.e., electrostatic force and hydrodynamic drag. The electrostatic force can be written as $F_{el} = qE,$ where q is the net charge on the particle and E is the applied field strength normalized by the electrical permittivity of DI water contaminated by the released counter-ion pairs from the coacervate. An increase in conductivity of DI water was detectable, but this increase was observed to be very low, and a function of the relative volume of the coaecervate droplers to the DI-water. The net charge q on a uniformly charged particle will be proportional to its surface area and can be written as $q = \alpha r^2$, where α is surface charge density of the particle (for positively charged particles, α is positive). The hydrodynamic drag on a particle moving with a velocity v can be simply modeled using Stokes' law, written as $F_d = 6\pi\eta r v,$ where η is the viscosity of the surrounding media. The equation of motion can be written as:

$$m\frac{d\boldsymbol{v}_{\boldsymbol{rel}}}{dt} = \alpha r^2\boldsymbol{E} - \beta r\boldsymbol{v}_{\boldsymbol{rel}}\,, \tag{6}$$

where, $\beta = 6\pi\eta$ is a constant for a given system and v_{rel} is the speed of the droplet with respect to the layer of fluid between the droplet and the solid coverglass on which the droplet is sedimented but not adsorbed. At long times, the droplet reaches its terminal velocity, and the net force on the droplet vanishes, giving:

$$\boldsymbol{v}_{\boldsymbol{rel}} = \frac{\alpha\boldsymbol{E}}{\beta}r\,. \tag{7}$$

Thus, the velocity of the droplet is proportional to its radius, with a slope proportional to the applied field strength, E.

Since the droplets are sedimented on a surface, there can be a field-strength-dependent drift from the surface underneath due to electro-osmotic flow. Moreover, due to the polarization of the droplet and associated electrohydrodynamic flows inside in presence of an external uniform field, there can be a polarization-induced drift of these droplets (we explain this phenomenon in the main text as well as in a later section below). The polarizability of these coacervates is still to be estimated quantitatively (see a later section below) and a rigorous measurement is beyond the scope of this paper. Given these two forces that can contribute to the drift of the droplet, it is probably coincidental that the data in **Fig. 3c** in the main text fits well with the equations described above. The drift in the droplet can thus be defined as coming from an "apparent charge density, α'" on the droplet which can be different from the actual charge density on the droplet.

Dielectric Polarization of Coacervate Droplets

The electric polarization $\boldsymbol{p}$ (or simply polarization for the rest of discussion) of a particle (or a droplet as in our case) of radius r_d in an external electric field, $\boldsymbol{E}$ can be expressed as,

$$\boldsymbol{p} = \varepsilon_o \varepsilon_s \beta r_d^3 \boldsymbol{E} \ , \tag{8}$$

where, ε_o is the permittivity of free space, ε_s is the relative permittivity (or dielectric constant) of the solvent, and β is as a measure of the polarizability of the droplet of dielectric constant ε_p, and is expressed as,

$$\boldsymbol{\beta} = \frac{\varepsilon_p - \varepsilon_s}{\varepsilon_p + 2\varepsilon_s} \ . \tag{9}$$

Since the polarization scales with the third power of particle size, larger droplets would have very high polarization. We will discuss the consequences of the size mismatch later. For now, let's try to understand how dipoles behave when they are in the vicinity of each other. The electric field $\boldsymbol{E_d}$ generated by a stationary dipole is given by,

$$\boldsymbol{E_d} = \frac{1}{4\pi\varepsilon_o\varepsilon_s} \frac{3(\boldsymbol{p}.\hat{\boldsymbol{r}})\hat{\boldsymbol{r}} - \boldsymbol{p}}{r^3} \ , \tag{10}$$

where $\boldsymbol{p}$ is the dipole moment of the droplet under consideration, **r** is the distance from the center of the droplet radially outward. If another dipole is present in the electric field of this dipole, it would feel a force of interaction. In general, the potential energy of a dipole in an external electric field is given by,

$$U = -\boldsymbol{p}.\boldsymbol{E} \ . \tag{11}$$

Hence, the interaction potential energy between two dipoles $\boldsymbol{p_1}$ and $\boldsymbol{p_2}$ separated by a center-to-center distance r is given by,

$$U_{12} = -\boldsymbol{p}_2.\boldsymbol{E}_1 = -\boldsymbol{p}_1.\boldsymbol{E}_2 = -\frac{1}{4\pi\varepsilon_o\varepsilon_s} \frac{p_1 p_2}{r^3} (3cos^2\theta - 1) \tag{12}$$

where θ is the angle between the vector connecting the two droplets and the electric field direction.

Dipolar Interaction of Droplets

The equations given above for dielectric polarization can have following consequences:

a) Position of the droplets with respect to each other governs interaction:

There is a direct relationship between the angle θ with the type of interaction between the two (dipole) droplets in an external electric field and can be understood as follows:

a. The potential energy is maximum when $\theta = 90°$. Here in this configuration, the droplets are side by side and repel each other. This repulsion leads to drifting of droplets away from each other.

b. There is a gradual change in the interaction between the droplets as the angle between the droplets decreases from $90°\ to\ 0°$. The repulsion between the droplets vanishes at an angle of $54.74°$ and the droplets start to attract each other at further lower angles. This angle is termed the *critical angle*. Small particles with thermal diffusion in the repulsive zone can easily go along this gradient and find themselves in the attractive zone.

c. The potential energy is minimum when $\theta = 0°$. This is the situation when the droplet dipoles are aligned head to tail, parallel to E-field. Here the attraction between a given droplet pair is maximum, and droplets attract each other strongly, giving rise to the chains which grow and align parallel to the external field lines.

These results of position-based interactions can be visualized in **Fig. S14**, where the center-to-center distance and the corresponding angle with external field line are plotted for two pairs of droplets as shown in **Fig. 4c** and **Video S7**. In the first case, the droplets P1-P2 were at an angle of $\approx 45°$ (with respect to the field lines) which was already less than the critical angle of $\approx 55°$, and thus when the field was turned on, the center-to-center distance decreased (with some fluctuations) until the droplets touched each other. In the second case, which is more interesting, the droplets P2-P3 initially are at an angle greater than the critical angle. Thus, upon turning on the electric field, the angle decreases (with some fluctuations) while the distance between the droplets fluctuates. Upon crossing the critical angle, the droplets started attracting each other and continued to move towards each other until they touched. In both cases, the droplets continued to align even after touching to minimize θ (and the potential energy). We think that the droplets are rolling with respect to each other during this alignment process.

b) Chain bundling at long times:

It may seem that different chains should repel each other when they are side by side as the droplet does so. However, this is not true, and in fact, at long times, we see side-by-side bundling of chains (**Fig. 4a**). This can be understood by considering the long-range behavior of the interaction potential of a droplet in one chain with all the droplets in other chains; even

though there is repulsion faced by a droplet from a few nearby chained droplets, there is attraction from droplets situated far off in the chains. Here, upon careful analysis, one would find that the attractive interactions dominate over the repulsive ones, and thus the bundling of chains to form columns is favorable. Moreover, at long times, the fluctuations in the dipolar electric field due to the thermal motion of the columns lead to interactions between them, contributing to this coarsening.

c) Droplet size governs clustering locations:

The interaction potential between a pair of droplets is directly proportional to the polarization of droplets. Since the magnitude of polarization increases dramatically with the increase in size (see Eq 8), the pairwise attraction (or repulsion) between droplets would be significantly higher when larger droplets are involved. Moreover, as the particle size increases, the drag on the particle increases, impeding its movement, which also leads to a lower thermal diffusivity. Consequently, the clustering of dipolar droplets is biased around larger droplets where smaller droplets, whose thermal diffusion is higher, in the vicinity of a large droplet are attracted towards it. In the initial chain formation process, larger droplets do not move much for the same reason and is quite evident in the time series images shown in **Fig. 4a**. This biased movement of droplets results in the formation of chains extending from larger droplets which are usually near the center, while smaller droplets are generally at the tails of the chains. Gast and Zukoski introduced a parameter λ that is a ratio of polarization energy to the thermal energy,[3]

$$\lambda = \frac{\pi \varepsilon_o \varepsilon_s r_d^3 (\beta E)^2}{k_b T} \quad . \tag{13}$$

Accordingly, larger particle size or higher field strength allows the droplets to overcome random thermal motion and take part in the chain formation process and was evident in our observations where chains of smaller droplets were found to be highly fluctuating (**Video S11**).

Droplet Clustering Kinetics

The cluster size distribution, $l_s(t)$, is usually defined as the number of chains of length s per unit area, at time t. This is used to calculate the average length of the chains $L(t)$, given by,

$$L(t) = \frac{\sum_s s^2\, l_s(t)}{\sum_s s\, l_s(t)}\,. \tag{14}$$

The number density of chains per unit area $n(t)$ at time *t* is given by,

$$n(t) = \sum_s n_s(t)\,. \tag{15}$$

where, $n_s(t)$ is the total number of clusters of size s.

Ion-segregation to Droplet Poles

Although in very small quantities, there is a natural presence of counter-ions in and around stabilized coacervate droplets. This was confirmed by the electrical conductivity of the continuous phase (prepared by centrifuging the solution to separate the droplets), which was around $106\ \mu S/cm$ corresponding to ≈ 0.1 to 1 mM NaCl. When dipoles are generated on droplets (or particles in general) in an external electric field, these solution ions tend to diffuse towards opposite polarity due to strong local electrostatic interactions with the poles. For e.g., Na^+ would diffuse towards the negative pole while Cl^- towards the positive. Upon reaching the poles successfully, these ions screen the dipolar interaction between the droplets leading to slower kinetics of chain formation. At high enough frequency of alternating electric field, the diffusion length scale, given by,

$$\delta l = \sqrt{Dt} \tag{16}$$

become much smaller than the droplet dimension, and thus these free ions do not reach the droplet poles, effectively giving a boost to chain formation kinetics.

Optimum Frequency for Chain Formation

There are two competing processes in the chain formation of particles that depends on frequency of the applied electric field. One is the movement of ions toward the droplet poles, which reduces the force between the droplets at low frequency.[3] This effect depends upon the nature of ions (their valance and their diffusivity), the distance that they need to travel when the E-field direction is switched (droplet size), and the conductivity of the continuous media (ion concentration). The other process is the reduction in polarizability of the polyelectrolyte chains at high frequency, which reduces the magnitude of the force between the particles.[4] This effect depends on the polarizability of polyions/macroions themselves in oscillating fields. Thus, an optimum frequency range would be required for the growth of chains of the particles.

Critical Field Strength and Polydispersity in Electrorheological Fluids

We plot our data for the chain length L(t) as a function of scaled time (τ_c) for a range of electric field strengths, we see in **Fig. S18** that a power-law scaling of $L(t)$ becomes established after a relatively short time. Here, we have chosen the theoretically reduced characteristic time (τ_c) predicted by the Halsey-Toor model ,[5]

$$\tau_c \sim (\frac{E}{E_o})^{4/5}t \quad . \tag{17}$$

Notably, a near "linear scaling" of (τ_c), consistent with the Halsey-Tool model, was observed in a previous experimental study of colloidal electrorheological fluids by Martin and coworkers.[6]

Our observations in the limit of a relatively large field strength evidently accord qualitatively with both the previous measurements of Martin and coworkers and Fraden and coworkers and with the scaling expectations of the Halsey-Toor model and that of Miguel and Pastor-Satorras.[5-8] Martin and coworkers found the chain coarsening exponent to be near 0.6 when a variable particle concentration was considered. This coarsening exponent is to be contrasted with the value of 0.33 from the spinodal decomposition of a phase separating mixture with short-range interactions. The Halsey-Toor model predicts a coarsening exponent of 5 / 9 = 0.555, which agrees with the estimate of this exponent by Martin and coworkers for colloidal electrorheological liquids and our own measurements on coacervate droplet suspensions at relatively high E-field strengths within experimental uncertainties. We find this consistency to be remarkable, given the rather simplified nature of the Halsey-Tool model and the rather different physical nature of the electrorheological fluids being compared in the present work.

It is also apparent from this figure that the curves for L(t) do not collapse so well at low field strengths. What might be different at low field strengths? First of all, the chains apparently do not form at all unless the E-field is "sufficiently large". This situation was suggested before by Fraden et al., but this possibility was not investigated systematically, as far as we know.[7]

It is worth recalling that in the case of ordinary liquid-liquid phase separation, the coarsening structures do not exist unless the temperature is changed from values in a range in which the fluid mixture is homogeneous to a temperature regime defined by the critical temperature for phase separation. Correspondingly, by analogy, we may expect a critical electric field strength for the initiation of our non-equilibrium electrorheological fluid phase separation process. However, this expected critical field strength, and the factors on which it might depend, have apparently not yet been determined in electrorheological fluid systems.

Beyond the matter of even the existence of chains of particles even at indefinitely long times, depending on whether the field strength is sufficiently high, there are additional issues about the chain coarsening process that could conceivably arise, which might spoil the 'universal' scaling of L(t) with time. In particular, the inherent size polydispersity of the stabilized droplets formed by our current vortexing method could be a significant factor in the coarsening dynamics when the field strength is large enough to ultimately cause some droplet string formation, but where at the same time the field response of particles of different size can significantly influence the rate of string formation. At this point, it is worth noting that the polarizability of a spherical droplet scales

with its volume, or the third power of its radius, and to the second power of the applied field. Smaller droplets in our highly polydisperse droplet dispersion might not participate to the same degree in chain formation because the critical strength for chaining is higher for these particles. This is just a hypothesis, but one that can be tested.

Motivated by these qualitative considerations, we then performed measurements on smaller droplets having a mean droplet size of 1 μm (compared to 4.7 μm in original experiments, **Fig. S20**) with a maximum droplet size of 2.5 μm (compared to 18 μm in original experiments) at a field strength of 14.3 V/cm (we isolated these small droplets by centrifuging the original DI water stabilized droplet emulsion at low speeds, allowing bigger droplets to sediment faster and leaving smaller droplets in suspension). The smaller droplets indeed did not form chains when an electric field was applied, even when left for a period of 30 min (shown in **Fig. S17**). It is noted that the area fraction, ϕ, in this experiment was ϕ = 0.14, a value much larger than the original experiments (ϕ = 0.4 to 0.8), which should, if anything, enhance the rate of chain formation.[8] These results provide support to our hypothesis that some of the non-universality in the low field coarsening kinetics as seen in **Fig. S18** derives from polydispersity of the droplets and the necessity of critical field strength to initiate chain formation that depends on particle size. The relative importance of these factors could be addressed by preparing the coacervate droplets in a more controlled way, e.g., using microfluidic methods to eliminate the polydispersity. Such methods would be very helpful in establishing and quantifying the suggested critical field strength for chain formation. These developments will also require additional measurements that shall be addressed in near future.

Dielectric Constant Estimation for Stabilized Coacervate Droplets

We mention in the main text that we might expect greater droplet stabilization with increasing charge, but this expectation can be contravened when the charge becomes so large that the counter-ions 'condense' on the polymers,[4, 9-14] spherical particles such as charged droplets,[14, 15] sometimes significantly altering particle permittivity and conductivity. This phenomenon has been studied in depth in the important context of cells[16, 17] and various biological materials composed of cells.[18-20] These observations in literature greatly complicate the modeling and characterization of highly charged colloidal suspensions because the charge and other properties are heavily renormalized by the dynamic counter-ion cloud in a way that depends on solution conditions (salt type, concentration, presence of boundaries, etc.). The polyelectrolyte components of many coacervate droplets, including our own, are themselves often highly charged and exhibit diffuse counter-ion clouds, and we may correspondingly expect these droplets to have high relative permittivity compared to water, as often observed in the case of cells and highly charged polyelectrolytes.[13, 14, 21] With this phenomenology of diffuse counter-ion layers tending to give rise to high relative permittivity of charged polymers droplets and particles in aqueous salt solutions in view, we now analyze the permittivity properties of our coacervate droplets.

Based on the kinetics of chain formation of stable coacervate droplets, even at low field strength compared to studies done with polystyrene particles in water,[7] it can be concluded that the magnitude of polarizability constant β of the coacervate-water system is very high, and thus there should be a high dielectric constant mismatch between the coacervates and DI water, the value for the latter (ε_s) being ≈ 80.[9] As noted above, we may anticipate the permittivity of our coacervate droplets and their associated counter-ion layers to be relatively large in comparison to water. Our preliminary observations based on dielectric spectroscopy of the coacervate macrophase indicate an apparent permittivity on the order of 10^3 to 10^4 in the frequency range of 10^2 to 10^5 Hz. This permittivity range is typical of systems such as DNA, cells, and other charged particle systems noted in the above general discussion, when normalized with respect to the concentration of polyelectrolyte in the coacervate (≈ 30 mass%, **Fig. S8**) as it has been found that the permittivity in polyelectrolyte solutions increases with increase in polyelectrolyte concentration.[4, 10, 11] We acknowledge that some previous studies of coacervates have indicated a much lower value, even lower than DI water.[22-24] We cannot account for this discrepancy, although we point out that these measurements were based on local fluorescence measurements that may not reflect the overall permittivity of the coacervate droplets. Our measurements, on the other hand, were performed on a macroscopic scale using dielectric spectroscopy, which probes the permittivity of the whole material. We infer from the strong field response of our coacervate droplets that they must be highly polarizable. In the future, we plan to better quantify this property and its frequency dependence in both the counterion-rich supernatant and the DI water.

Electrohydrodynamic Flows Inside the Stabilized Droplets under Uniform External Field

To visualize EHD flows, we prepared stabilized coacervate droplets by adding FluoSpheres™ Carboxylate-Modified Microspheres (from Thermo Fisher Scientific, USA) of 2.0 µm size into the ATP solution before PDDA addition, followed by vortexing of macrophase in DI water for stabilization of droplets. They were then subjected to a ac e-field of 14.3 V/cm in strength at a frequency of 100 mHz. A 100 mHz frequency has a time period of 10 s, so that the droplet moves in one direction for 5 s, and then in the reverse direction for the next 5 s, and so on.

We found that our DI water stabilized droplets exhibited convective flows in the dc field during translocation. The flow in the droplet appeared to be in bilateral symmetry, and the flow in the center of the droplet was in opposite direction to that of droplet motion (see **Video S13**). We are brief in our description of electro-convective flow in our coacervate droplets since we found an excellent and extensive account of this phenomenon by Yin et al.,[25] and because our main intent of including these observations in the present work was simply to demonstrate that the interior of the coacervate droplets is in a fluid-like state.

The measurements of Yin et al. were for coacervate droplets in their equilibrium, counterion-rich supernatant. The EHD flows inside the droplet would also appear outside it in the surrounding water. Since the droplet is sedimented on the surface, the flows around the droplets can be asymmetric. These flows may generate a drift on the droplet of their own, and they can support or counter the charge-based drift of the droplet.

Supplementary Tables:

Polycation	Polyanion	In counterion-rich supernatant	In de-ionized (DI) water
PDDA	ATP	**Coacervate**	**Droplets**
″	CMDex20	**Coacervate**	**Droplets**
″	CMDex150	**Coacervate**	**Droplets**
″	HA15	**Coacervate**	**Droplets**
″	HA80	**Coacervate**	**Precipitate**
″	PSS	**Precipitate**	----
PDDA240	ATP	**Coacervate**	**Droplets**
″	CMDex20	**Coacervate**	**Droplets**
″	CMDex150	**Coacervate**	**Droplets**
″	HA15	**Coacervate**	**Droplets**
″	HA80	**Precipitate**	----
″	PSS	**Precipitate**	----
εPL	ATP	**Coacervate**	**Droplets**
″	CMDex20	**Coacervate**	**Droplets**
″	CMDex150	**Coacervate**	**Droplets**
″	HA15	**Coacervate**	**Droplets**
″	HA80	**Coacervate**	**Precipitate**
″	PSS	**Precipitate**	----

Table S1: **Mixtures of polycations and polyanions leading to coacervation or precipitation in their counterion-ion rich supernatant and their behavior in DI water upon macrophase transfer.** PDDA240 denotes a high molecular mass variant of PDDA (see list below). Here, the strong polyanion polystyrene sulfonate (PSS) formed precipitates with all the polycations. Upon increasing the molecular mass of hyaluronic acid (HA), precipitates were formed with the high molecular mass variant of PDDA. Furthermore, while most of the coacervates formed droplets upon macrophase transfer to DI water, the high molecular mass HA formed precipitates.

List of polymers used here:
PDDA : poly (dimethyldiallylammonium chloride), 8.5 kDa
PDDA240 : poly (dimethyldiallylammonium chloride), 240 kDa
εPL : poly epsilon L-lysine hydrochloride, 3.5-4.5 kDa
ATP : adenosine triphosphate disodium salt hydrate
CMDex20 : carboxymethyl dextran sodium salt, 20 kDa
CMDex150 : carboxymethyl dextran sodium salt, 150 kDa
HA15 : hyaluronic acid sodium salt, 8-15 kDa
HA80 : hyaluronic acid sodium salt, 70-80 kDa
PSS : sodium poly (styrene sulfonate),70 kDa

Supplementary Figures:

	PDDA	PDDA240	εPL
**ATP**	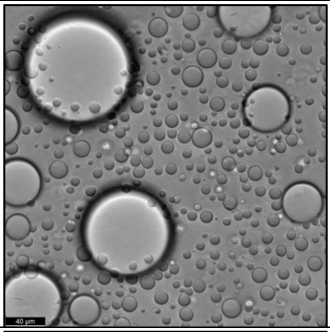	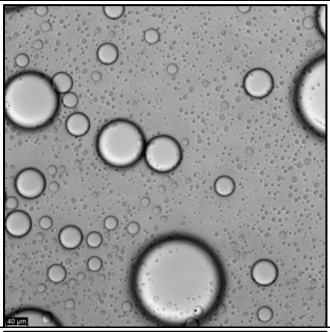	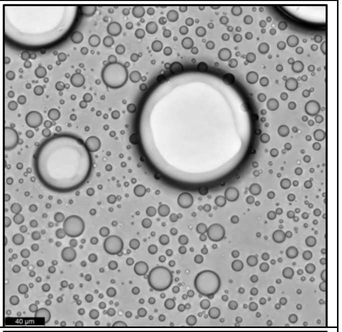
**CMDex20**	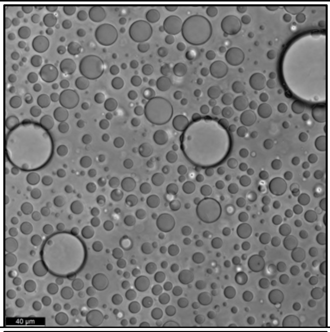	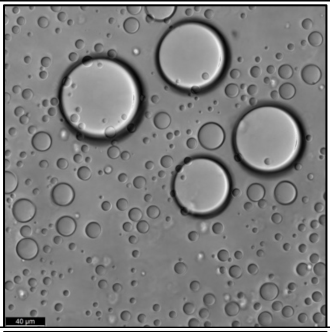	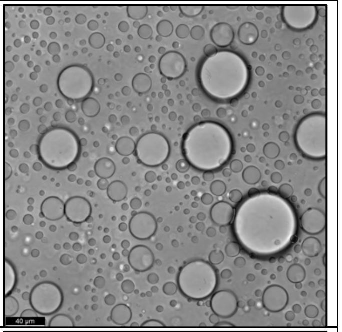
**CMDex150**	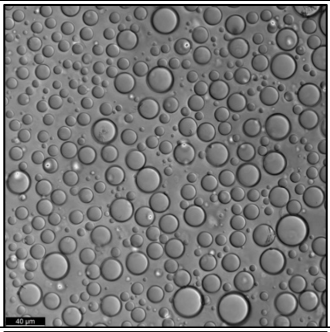	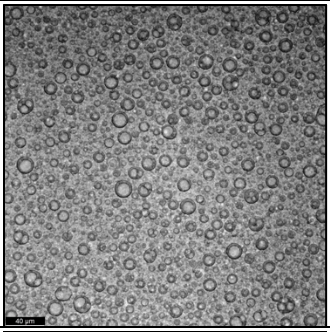	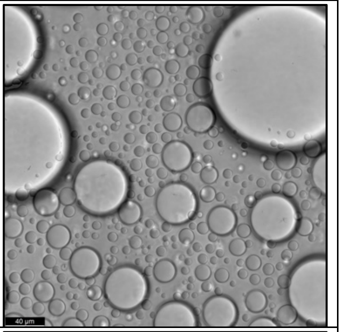
**HA15**	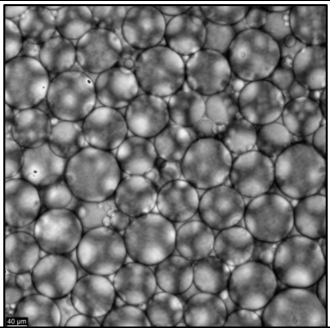	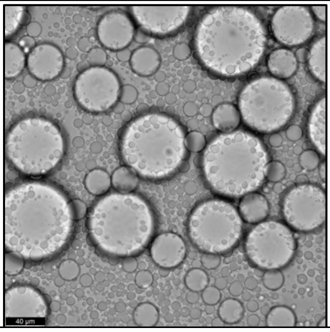	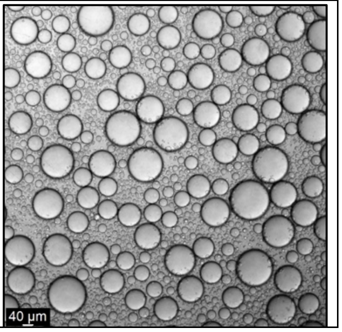

**HA80**	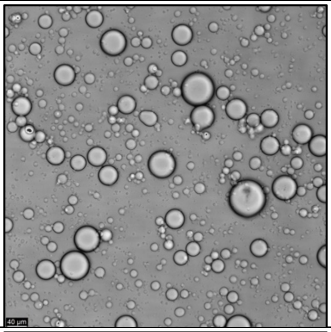	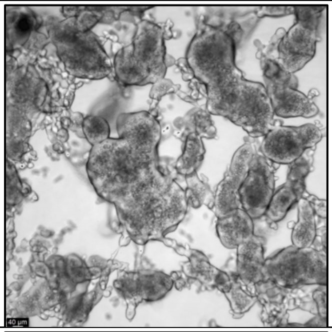	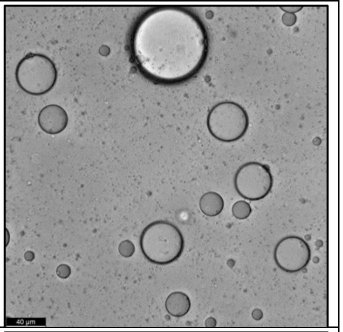
**PSS**	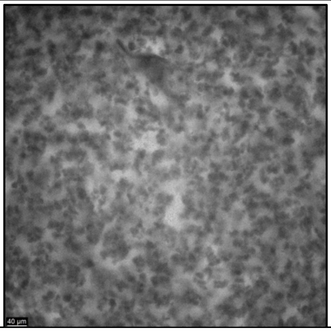	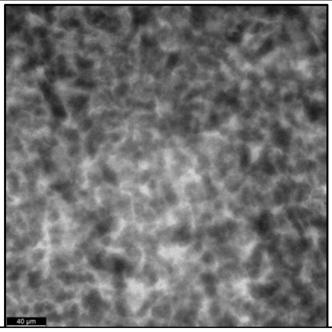	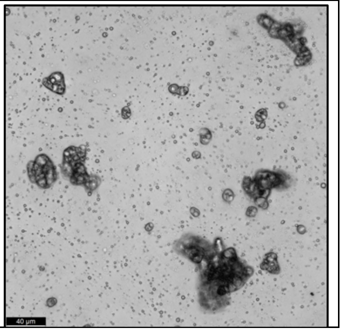

Figure S1: Brightfield microscopy images of coacervate/precipitates formed upon mixing polyelectrolytes of opposite charges. Here, experiments were done without any exogenous addition of salt ions. The scale bar for each image is at the bottom left and is equal to 40 μm.

(a)

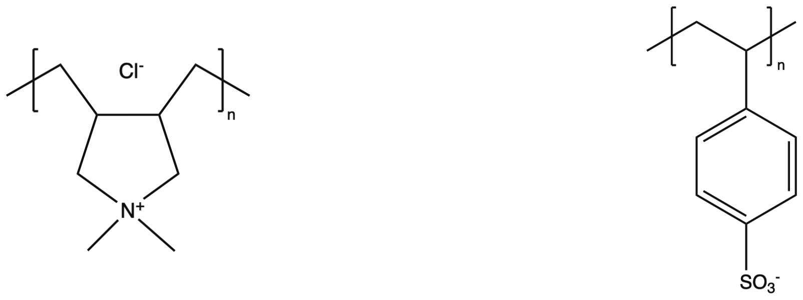

(b)

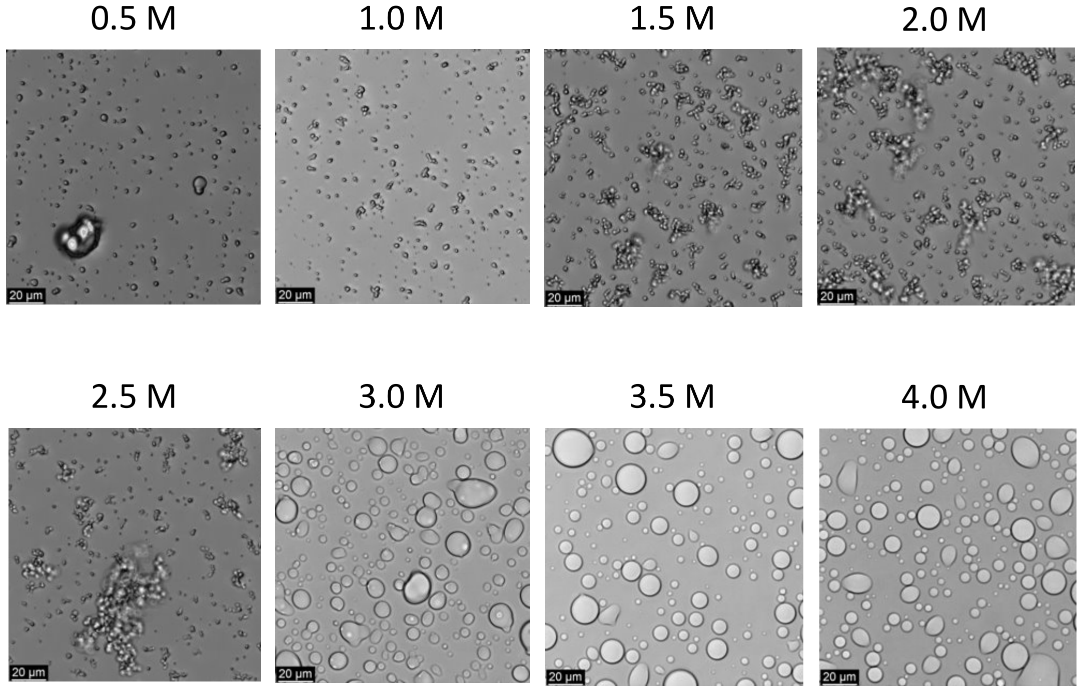

Figure S2: PDDA-PSS forms solid precipitate when mixed, and exogenous salt ions were required for droplet formation. (a) Structures of PDDA and PSS. (b) Bright-field microscopy images showing precipitates to coacervate transition for PDDA-PSS polymer combination at different salt concentrations (0.5 M to 4.0 M NaCl). The transition was found to happen between 2.5 M and 3.0 M NaCl.

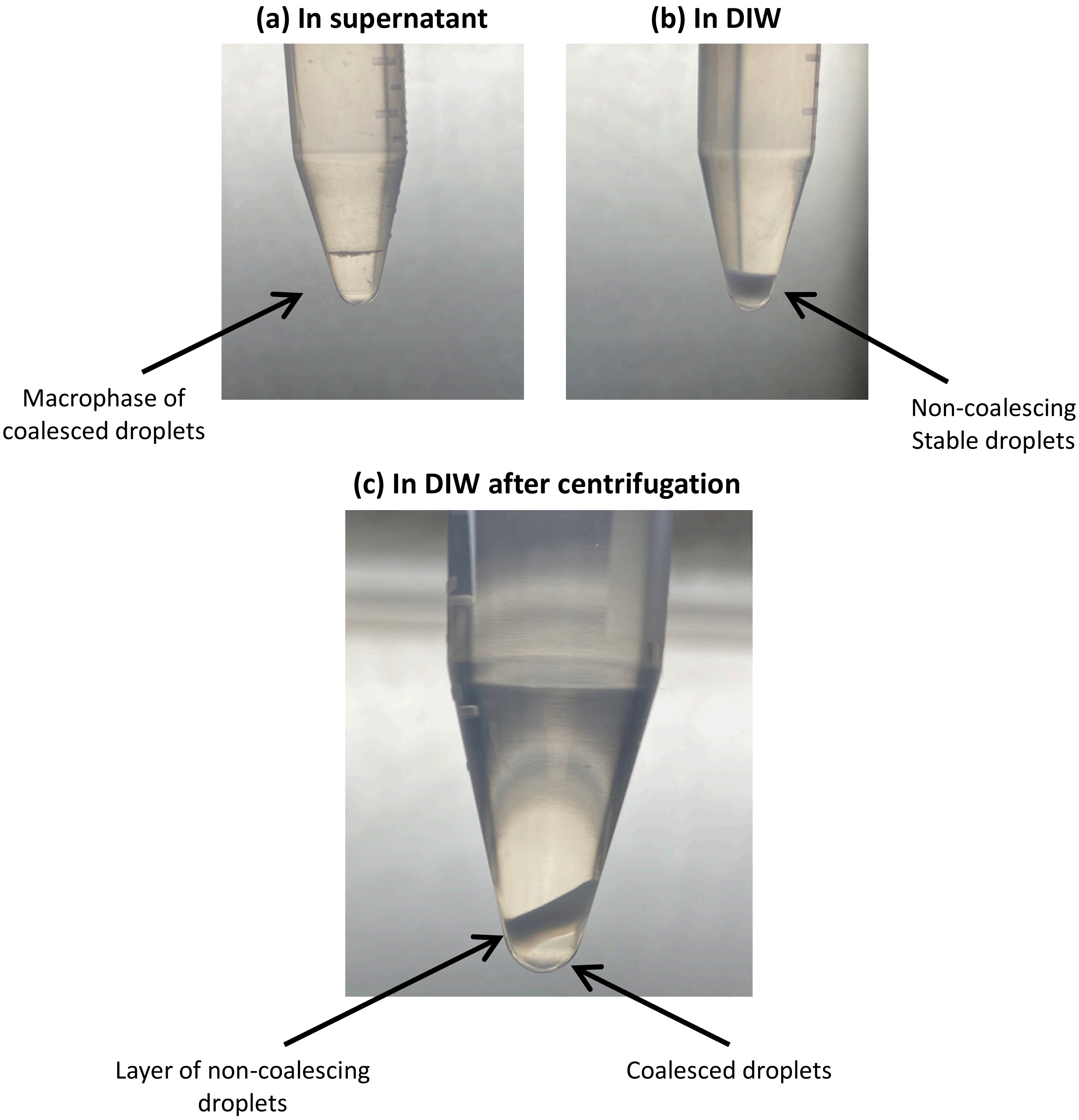

Figure S3: Photographs showing coacervate sample after centrifugations in vials. (a) Droplets in counterion-rich supernatant macrophase separated in layers after 5 min of 2000 x g. (b) Stable droplets in DI water did not coalesce after 10 min of 2000 x g. (c) When centrifuged at 5000 x g for 30 min, some of the stable droplets in (b) do coalesce to form a macrophase separated material.

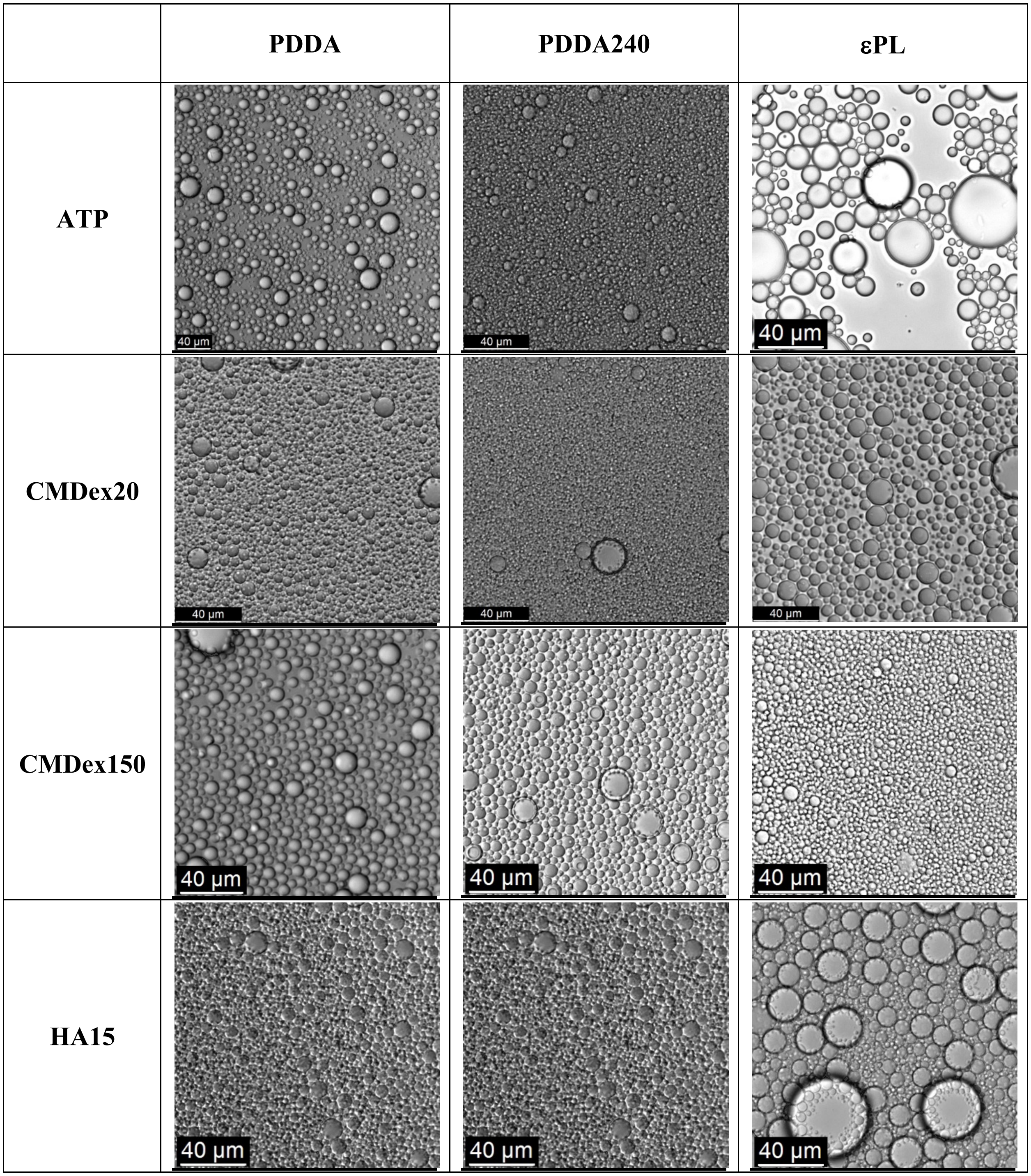

Figure S4: Brightfield microscopy images of droplets formed upon transferring and vortexing the coacervate macrophase to DI water from their counter-ion rich supernatant. The images were taken 48 h after the sample preparation.

(a)

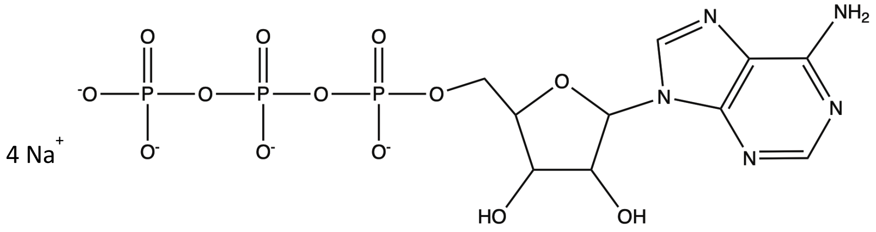

(b)

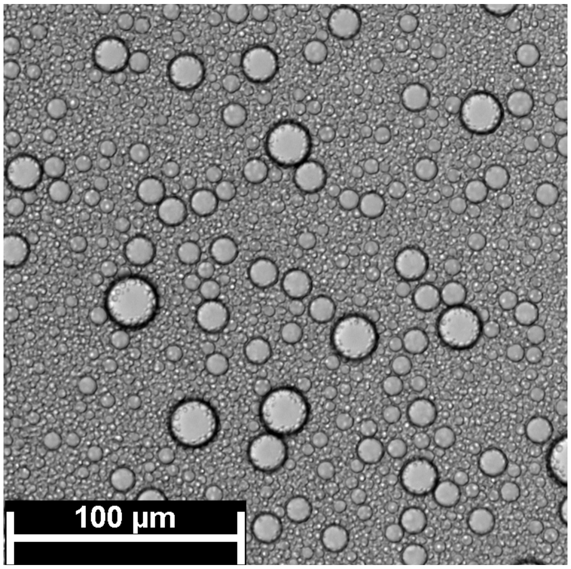

Figure S5: Droplet stability of PDDA-ATP coacervates in DOI water. (a) Molecular structure of ATP with sodium cation. (b) Bright-field microscopy image showing DI water redispersed PDDA-ATP coacervate droplets after a month of incubation at standard lab conditions.

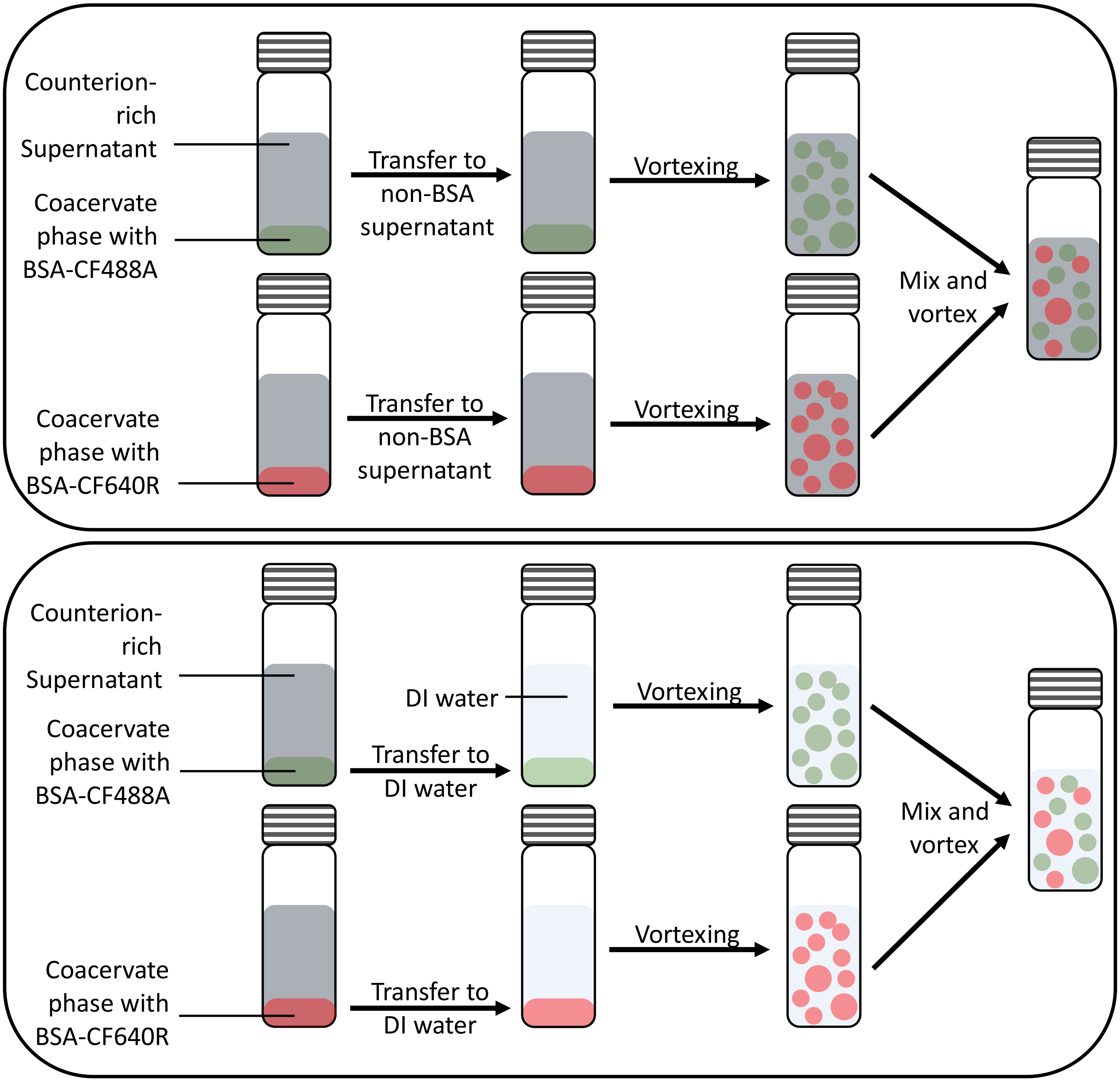

Figure S6: Illustrations of mixing schemes. Top: Mixing of droplets in counterion-rich supernatant. Here individual macrophase was first transferred from respective supernatant to BSA-free supernatant to diffusion of BSA-CF488A from supernatant to droplets containing BSA-CF640R and vice-versa. Bottom: Mixing of droplets in DI water. Here individual macrophase was first transferred to DI water to make stable droplets, and the stable droplets were then mixed.

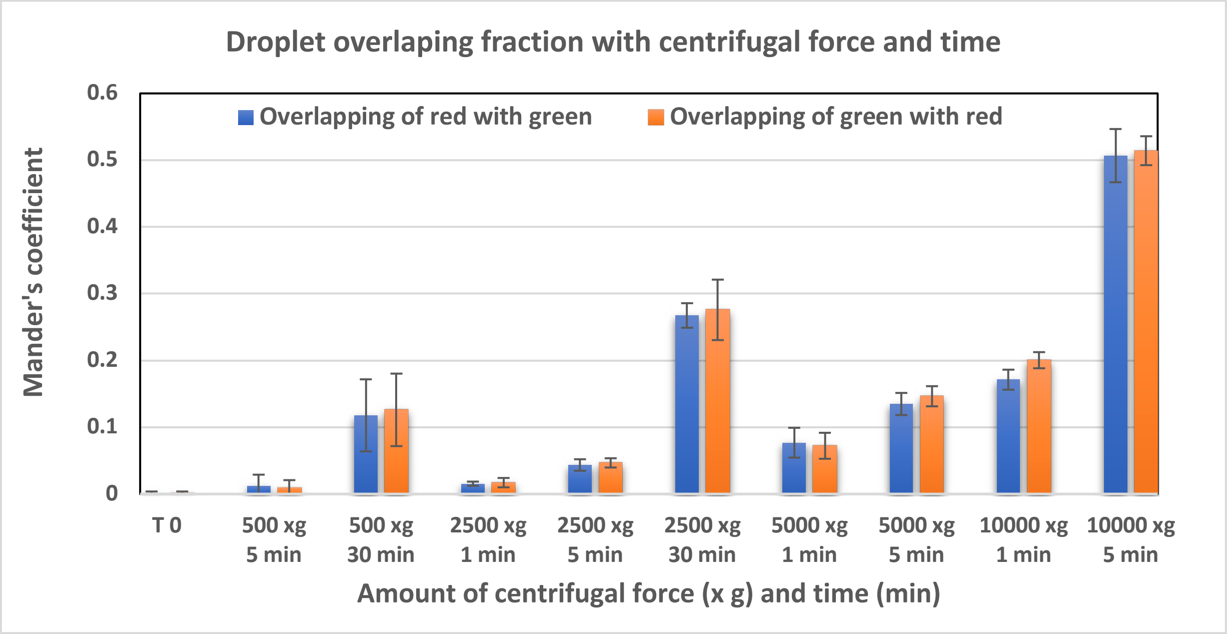

Figure S7: Plot of Mander's coefficient (an indicator of droplet coalescence) with centrifugal speed and time for BSA-CF488A and BSA-CF640R encapsulated stable coacervate droplet of PDDA-ATP. The plot signifies the stability of droplets even under applied external pressure. See supplementary notes 1 for more details. The error bars denote the standard deviation in 9 different image sets.

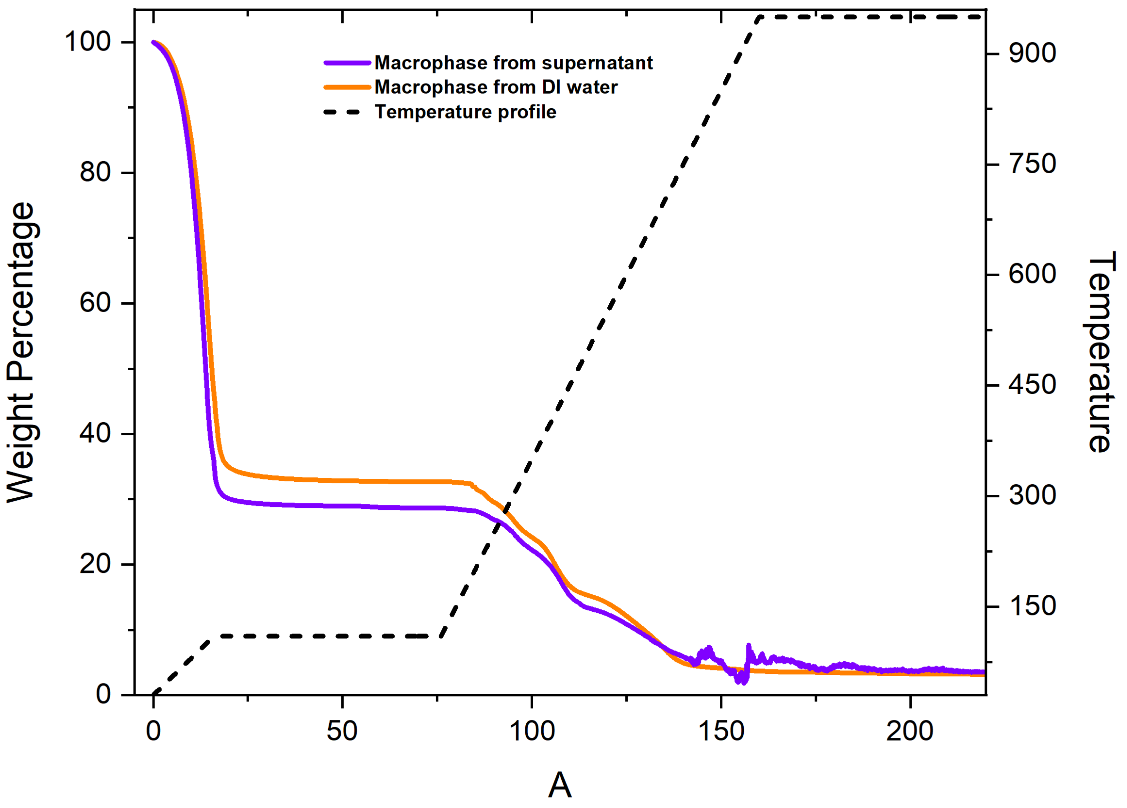

Figure S8: Determining water content of the coacervates in different environments. Mass loss of coacervate macrophase taken from the counter-ion rich supernatant (violet) and the deionized water (orange) upon heating inside a furnace (thermogravimetric analysis). The temperature profile is shown by the black dashed line. The temperature is maintained at 110 °C for an hour to remove all the water from the sample to estimate its contribution to the overall mass of the sample. Using this data, we estimate the water content of coacervates from supernatant as 70% and coacervates from DI water as 65%.

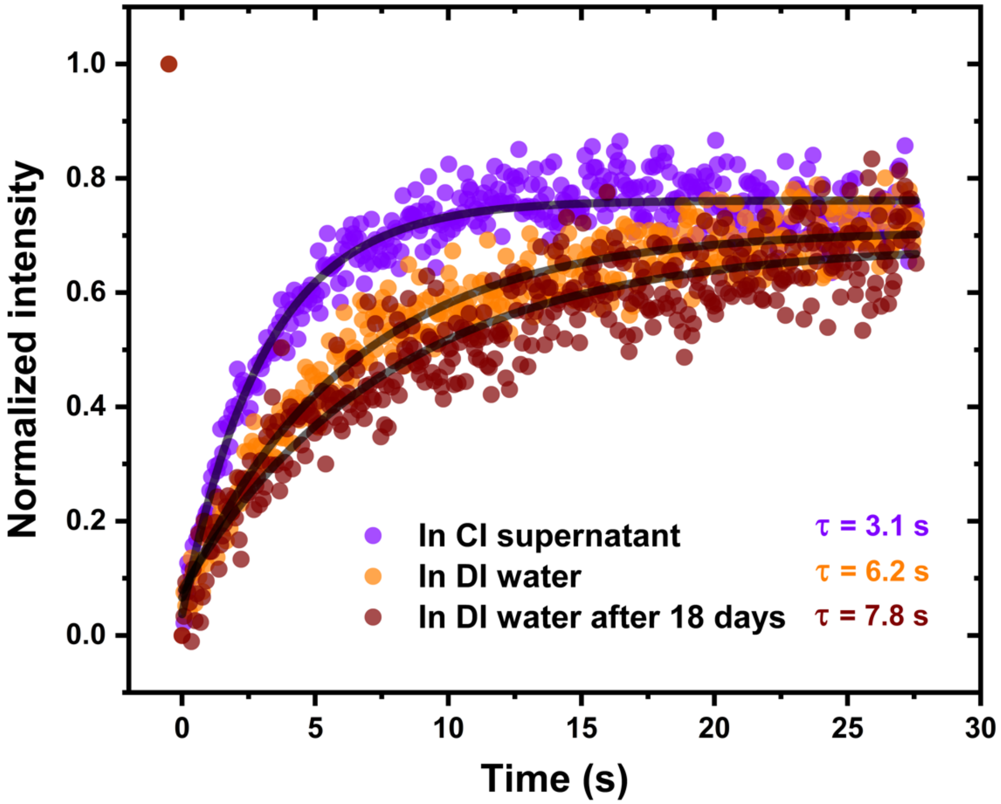

Figure S9: FRAP data for PDDA-ATP coacervate droplets. Extended plot of Fig. 2d. showing the recovery data for droplets stabilized in DI water after 18 days of incubation. CI supernatant represents counterion-rich supernatant.

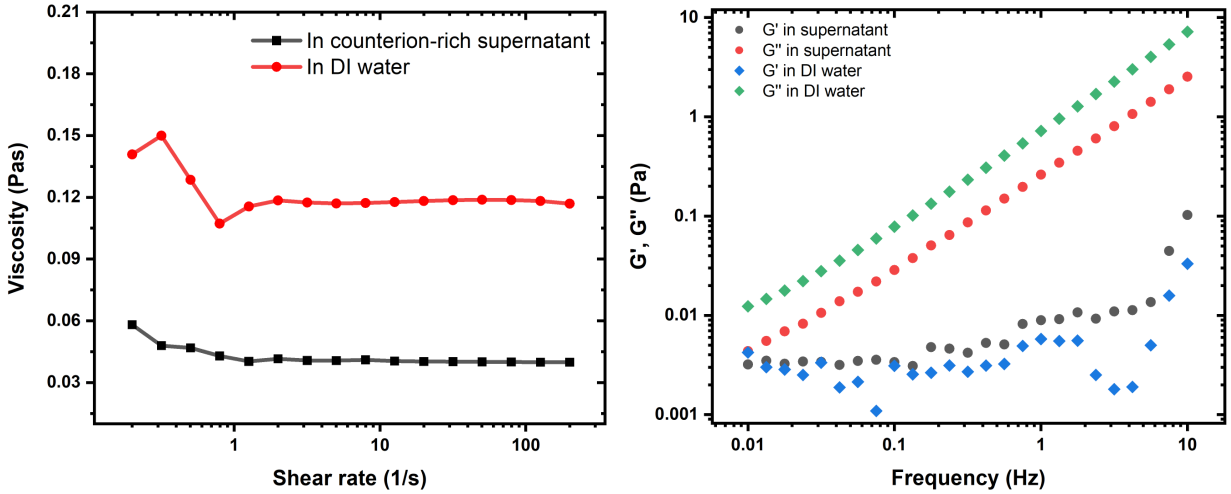

Figure S10: Bulk rheology data for PDDA-ATP coacervate droplets. Viscosity vs. strain rate (left) and *G'* (storage or elastic modulus) *and G"* (loss or plastic modulus) vs. frequency (right) plots for PDDA-ATP coacervate macrophases.

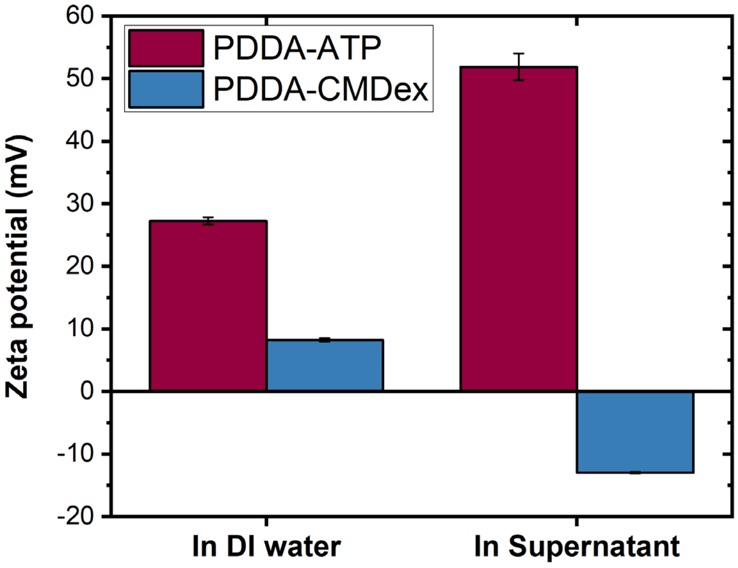

Figure S11: Plots of zeta-potential of coacervate droplets in DI water and in counterion-rich supernatant. The zeta potential of coacervates droplets decreases upon transfer to DI water for PDDA-ATP droplets. For PDDA-CMDex20 droplets, the zeta potential becomes slightly positive upon transfer to DI water. For both cases, however, the zeta potential values cannot explain the exceptional stability of coacervate droplets upon the macrophase transfer to DI water.

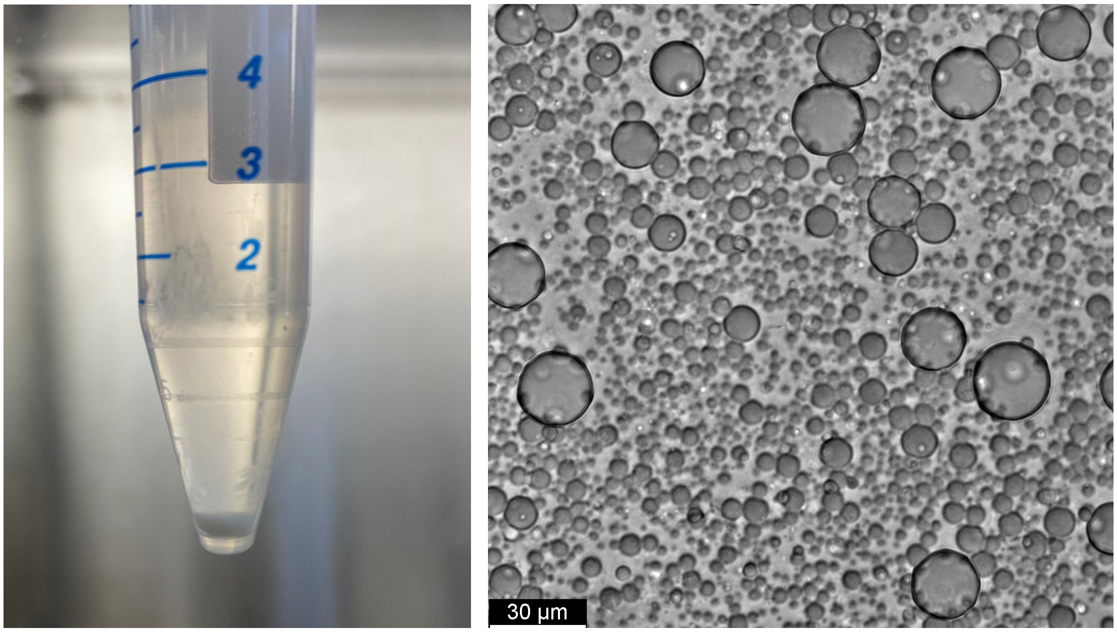

Figure S12: Long-term stability of droplets in DI water. Images of droplets stabilized and isolated for nearly two years in a tube left at standard lab conditions. (**Left**) Droplets sedimented at the bottom of the tube appear whitish, similar to the freshly stabilized sample shown in Fig. S3b, and do not macrophase separate even after 21 months of isolation. (**Right**) Bright-field image of the sample from the tube showing spherical droplets.

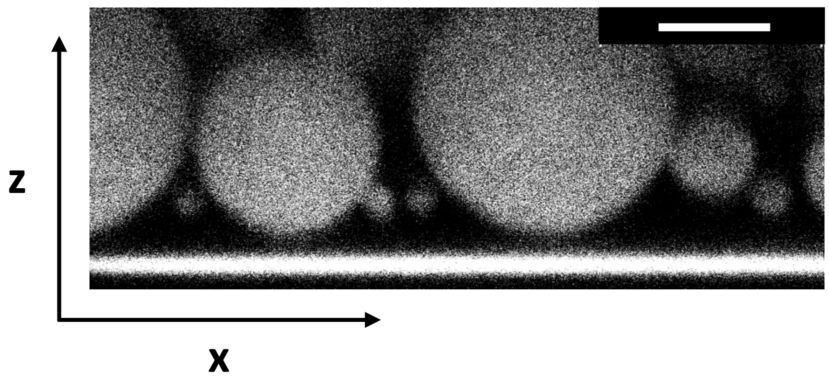

Figure S13: Droplets reform to spherical shape. Confocal microscopy image of stabilized coacervate droplets sedimented on coverglass upon redispersion in DI water after centrifugation induced deformation inside a thin capillary. The coverglass was coated with PVP as described in the methods section. Scale bar: 5 μm.

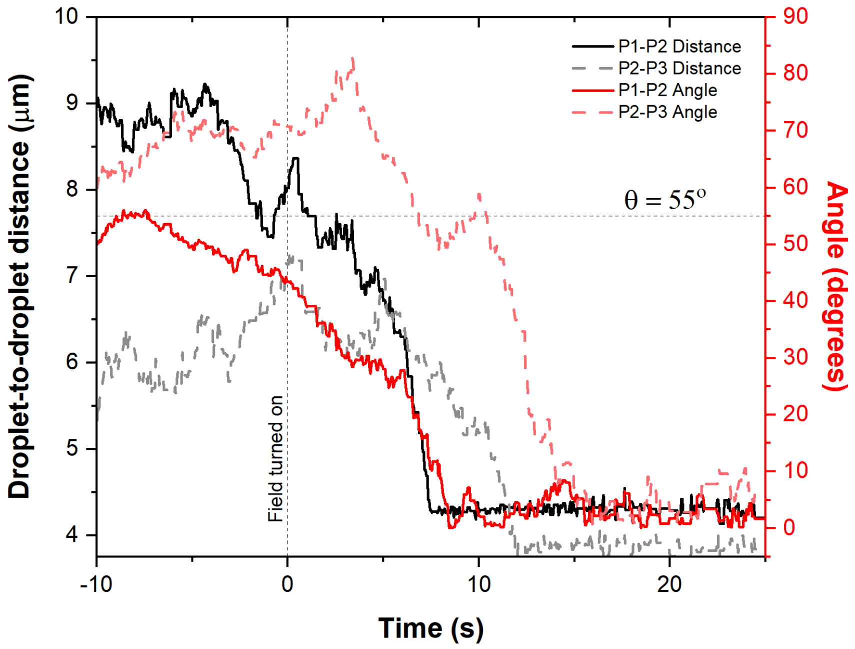

Figure S14: Plot of droplet-to-droplet distance and angle between them over time upon turning on the electric field. Here, two interdependent droplet pairs were examined, as shown in Fig. 4c. The field was turned on at t = 0, as shown on the plot.

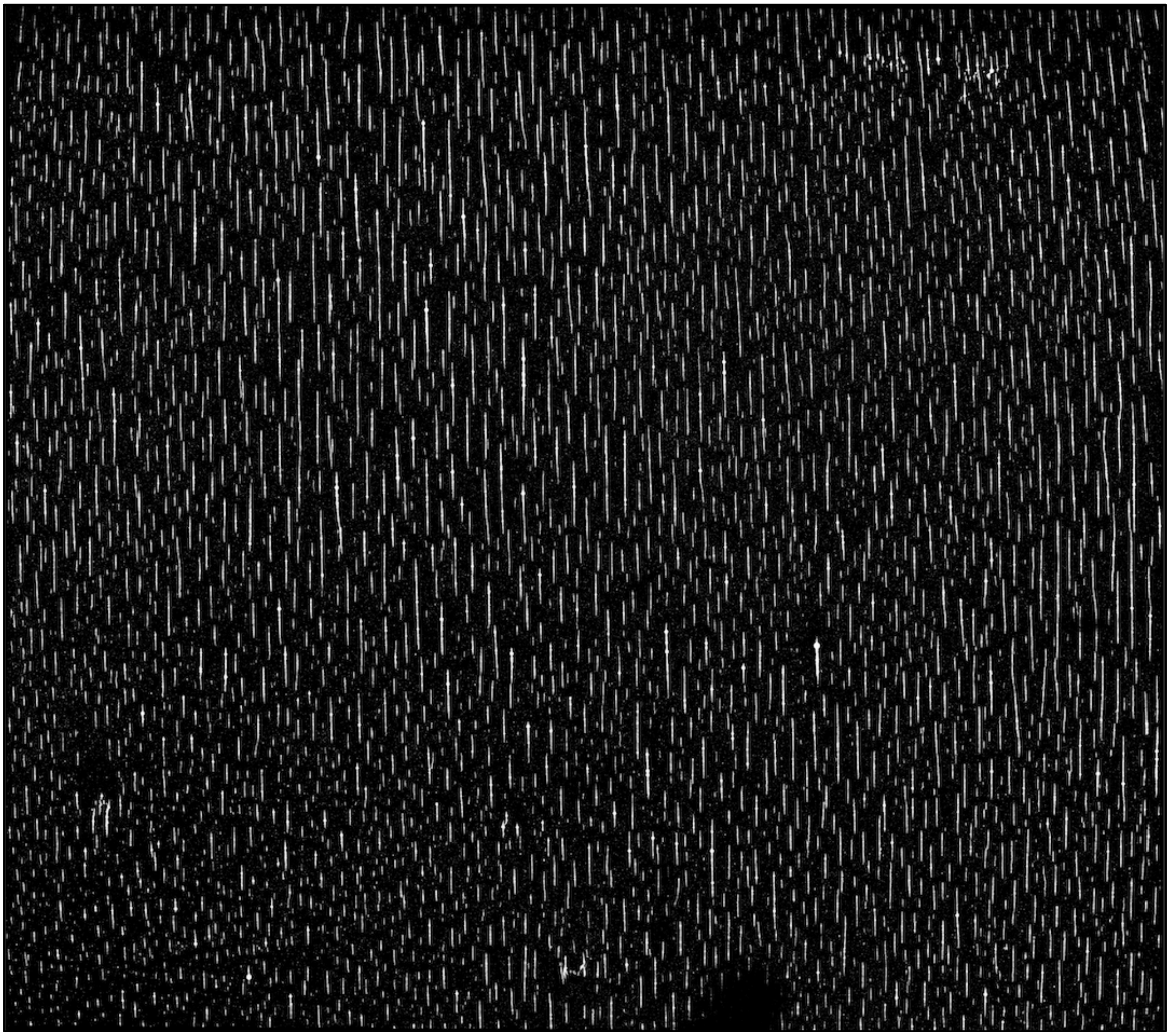

Figure S15: Large area droplet-chain patterning. Confocal microscopy image over an area of 4.2 mm x 3.7 mm, reconstructed by stitching smaller images, showing parallel chain pattern formed by coacervate droplets after 30 min of turning on an external field of 44.6 V/cm at 100 kHz.

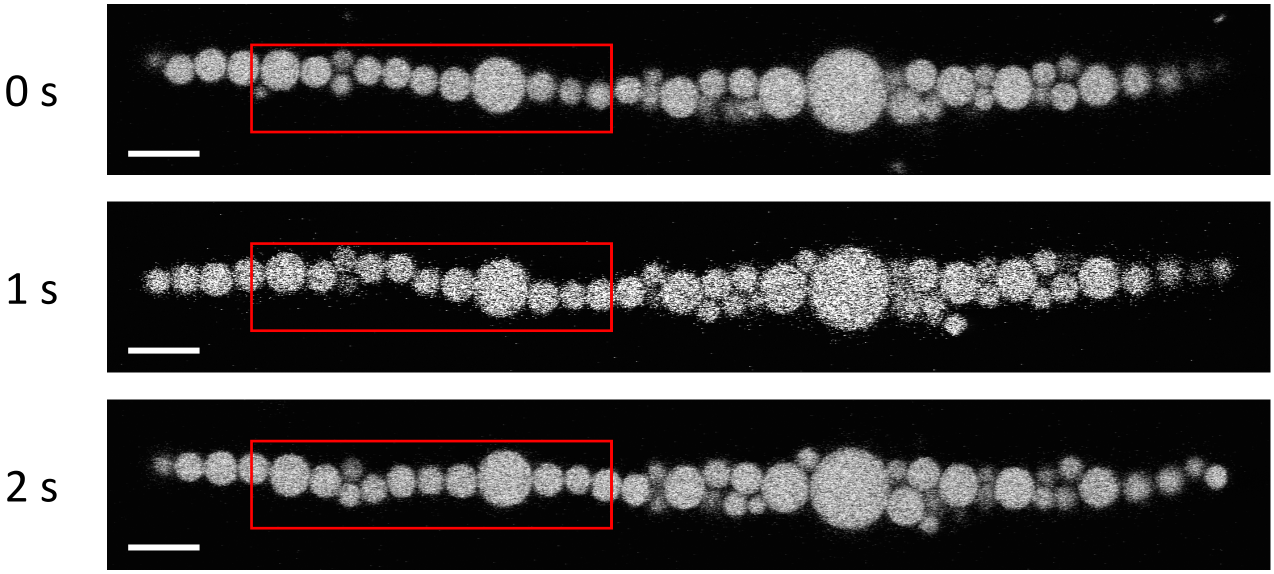

Figure S16: Confocal microscopy images showing fluctuations in a chain of coacervate droplets. Images were collected at 35.7 V/cm at 100 kHz at different times. The rectangle drawn highlights the fluctuations. Scale bar: 5 µm.

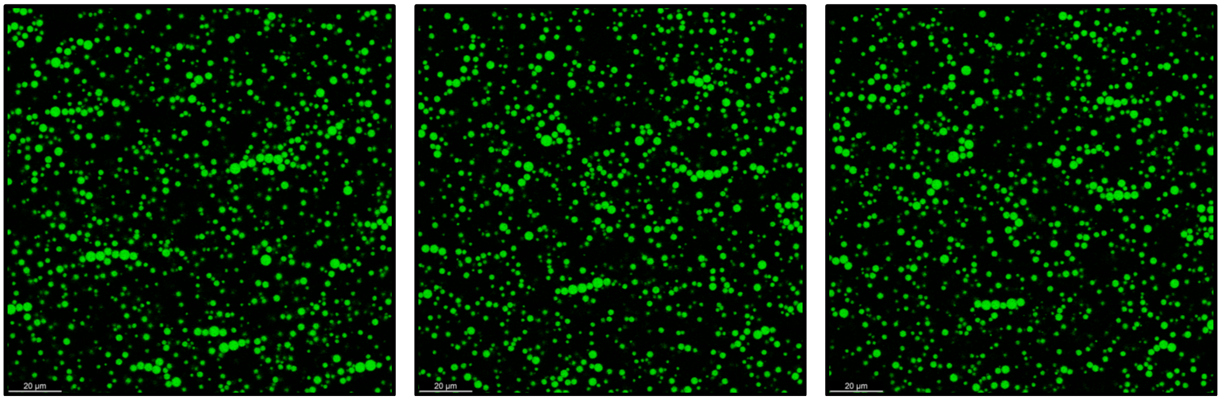

Figure S17: Confocal microscopy showing reversible clustering of small droplets at low electric fields. Small droplets (~ 1 μm average size) couldn't form long chains at low field strength (14.3 V/cm at 100 kHz). A few clusters that formed were found to dissolve over time. Images shown were taken 26 min (left), 28 min (center), and 30 min (right) after the field was turned on. Scale bar: 20 μm.

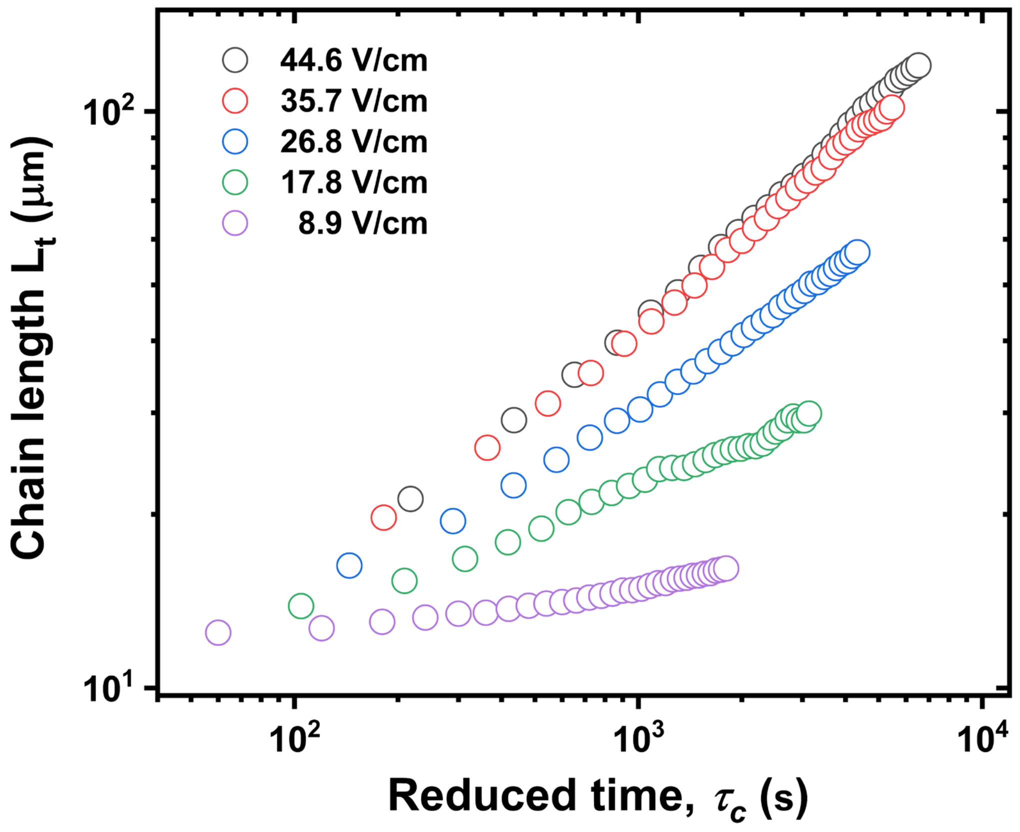

Figure S18: Asymptotic behavior of chain length (L_t) for different voltages with reduced time $\boldsymbol{\tau_c}$. The reduced time τ_c is defined in the Eq. (17) of SI Appendix.

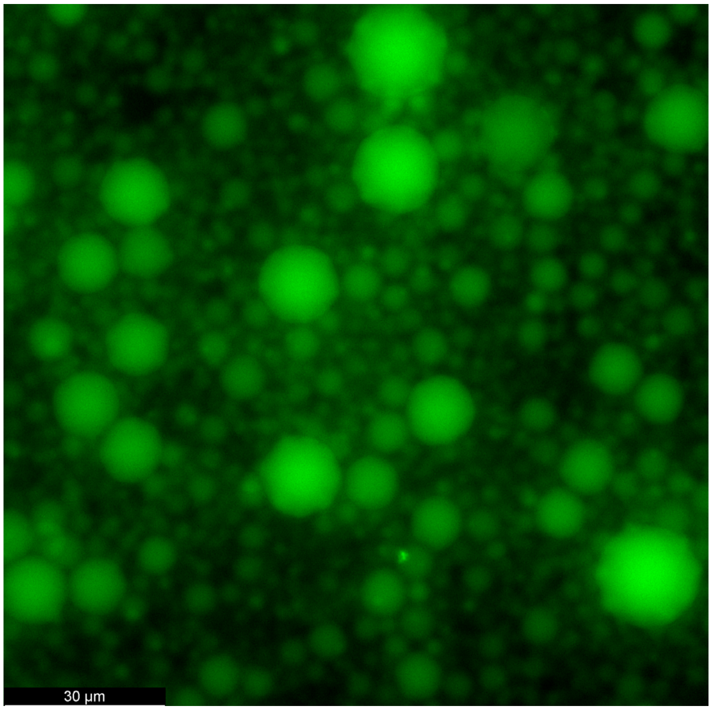

Figure S19: Fluorescence micrograph of resorufin inside coacervate droplets. An aqueous solution of a small uncharged molecule, Resorufin, was added to the stabilized coacervate droplet suspension. The small molecules diffuse into stabilized droplets after a very short time.

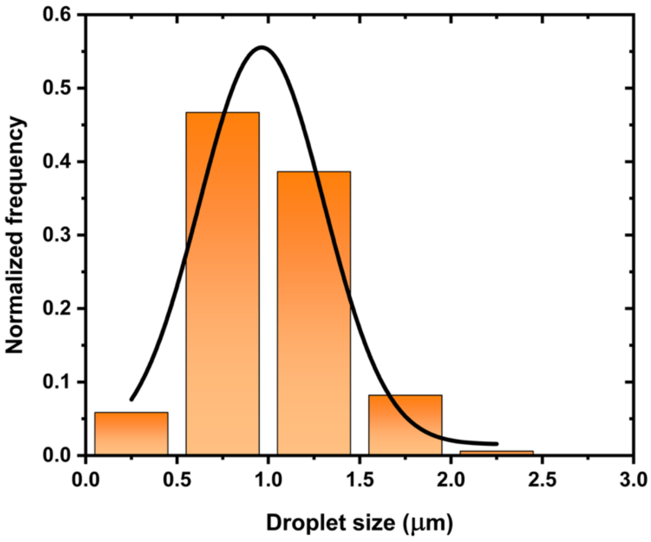

Figure S20: Droplet size distribution of smaller droplets of stabilized PDDA-ATP. These were separated from the rest by centrifuging and used for e-field study in Fig. S17. The average size of these droplets is $\approx 1.01\ \pm 0.34\ \mu m$.

Legends for Videos:

Video S1: Coalescence of PDDA-ATP coacervate droplets when suspended in equilibrium supernatant.

Video S2: Translation of stabilized PDDA-ATP coacervate droplets in straight lines in the presence of an external DC electric field of strength 13.7 V/cm. The direction of the field (positive to negative) is from left to right.

Video S3: Example #1 of controlled motion of a stabilized coacervate droplet along the characters *UH* (standing for University of Houston). The strength of field used here is 13.7 V/cm.

Video S4: Example #2 of controlled motion of a stabilized coacervate droplet along the characters *KRG* (standing for Karim Research Group). The strength of field used here is 21.4 V/cm.

Video S5: Formation of chains of stabilized coacervate droplets under an external AC electric field of 35.7 V/cm at a frequency of 100 kHz (from Fig. 4a).

Video S6: The breakup of chains upon turning off the external field (from Fig. 4b).

Video S7: Clustering of three isolated droplets upon turning on the external electric field of 17.9 V/cm at 100 kHz frequency.

Video S8: Translation motion of droplet chains under AC electric field with a DC bias. The strength of the AC field is 8.9 V/cm at 100 kHz, while the DC bias strength is 8.9 V/cm or -8.9 V/cm (for motion in the reverse direction). The direction of the field is horizontal.

Video S9: Levitation of stabilized droplets upon turning on the external E-field.

Video S10: Large area confocal videography of chain formation by stabilized droplets in the external field for statistical analysis.

Video S11: Fluctuations of droplets in a thin chain (fiber).

Video S12: Fluctuations of droplets in a thick chain (bundle).

Video S13: EHD flow inside coacervate droplets under ac e-field of 14.3 V/cm at 100 mHz. The 100 mHz frequency corresponds to a period of 10 sec. Hence, the direction of the E-field reverses after every 5 seconds, and so does the direction of the droplet movement. Correspondingly, the EHD flows also reverse.

References:

1. Bolte, S.; Cordelières, F. P., A guided tour into subcellular colocalization analysis in light microscopy. *Journal of Microscopy* **2006,** *224* (3), 213-232.
2. McCall, P. M.; Srivastava, S.; Perry, S. L.; Kovar, D. R.; Gardel, M. L.; Tirrell, M. V., Partitioning and Enhanced Self-Assembly of Actin in Polypeptide Coacervates. *Biophysical Journal* **2018,** *114* (7), 1636-1645.
3. Gast, A. P.; Zukoski, C. F., Electrorheological fluids as colloidal suspensions. *Advances in Colloid and Interface Science* **1989,** *30*, 153-202.
4. Bordi, F.; Cametti, C.; Colby, R. H., Dielectric spectroscopy and conductivity of polyelectrolyte solutions. *Journal of Physics: Condensed Matter* **2004,** *16* (49), R1423-R1463.
5. Halsey, T. C.; Toor, W., Structure of electrorheological fluids. *Physical Review Letters* **1990,** *65* (22), 2820-2823.
6. Martin, J. E.; Odinek, J.; Halsey, T. C.; Kamien, R., Structure and dynamics of electrorheological fluids. *Physical Review E* **1998,** *57* (1), 756-775.
7. Fraden, S.; Hurd, A. J.; Meyer, R. B., Electric-field-induced association of colloidal particles. *Physical Review Letters* **1989,** *63* (21), 2373-2376.
8. Miguel, M. C.; Pastor-Satorras, R., Kinetic growth of field-oriented chains in dipolar colloidal solutions. *Physical Review E* **1999,** *59* (1), 826-834.
9. Malmberg, C.; Maryott, A., Dielectric constant of water from 0 to 100 C. *Journal of research of the National Bureau of Standards* **1956,** *56* (1), 1-8.
10. Takashima, S., Dielectric Dispersion of Deoxyribonucleic Acid. II1. *The Journal of Physical Chemistry* **1966,** *70* (5), 1372-1380.
11. Tomić, S.; Babić, S. D.; Vuletić, T.; Krča, S.; Ivanković, D.; Griparić, L.; Podgornik, R., Dielectric relaxation of DNA aqueous solutions. *Physical Review E* **2007,** *75* (2), 021905.
12. Cole, R. H., Dielectric Theory and Properties of DNA in solution. *Annals of the New York Academy of Sciences* **1977,** *303* (1), 59-73.
13. Chremos, A.; Douglas, J. F., Polyelectrolyte association and solvation. *The Journal of Chemical Physics* **2018,** *149* (16), 163305.
14. Chremos, A.; Douglas, J. F., The Influence of Polymer and Ion Solvation on the Conformational Properties of Flexible Polyelectrolytes. *Gels* **2018,** *4* (1).
15. Sarojini, K. G. K.; Manoj, S. V.; Singh, P. K.; Pradeep, T.; Das, S. K., Electrical conductivity of ceramic and metallic nanofluids. *Colloids and Surfaces A: Physicochemical and Engineering Aspects* **2013,** *417*, 39-46.
16. Di Biasio, A.; Cametti, C., Polarizability of spherical biological cells in the presence of localized surface charge distributions at the membrane interfaces. *Physical Review E* **2010,** *82* (2), 021917.
17. Nasir, N.; Al Ahmad, M., Cells Electrical Characterization: Dielectric Properties, Mixture, and Modeling Theories. *Journal of Engineering* **2020,** *2020*, 9475490.
18. Pethig, R., Dielectric properties of body tissues. *Clinical Physics and Physiological Measurement* **1987,** *8* (4A), 5-12.
19. Gabriel, C.; Gabriel, S.; Corthout, E., The dielectric properties of biological tissues: I. Literature survey. *Physics in Medicine and Biology* **1996,** *41* (11), 2231-2249.
20. Gabriel, S.; Lau, R. W.; Gabriel, C., The dielectric properties of biological tissues: III. Parametric models for the dielectric spectrum of tissues. *Physics in Medicine and Biology* **1996,** *41* (11), 2271-2293.

21. Chremos, A.; Douglas, J. F., Communication: Counter-ion solvation and anomalous low-angle scattering in salt-free polyelectrolyte solutions. *The Journal of Chemical Physics* **2017,** *147* (24), 241103.
22. Zhuang, M.; Zhang, Y.; Zhou, S.; Zhang, Y.; Wang, K.; Nie, J.; Liu, J., Uricase-containing coacervate microdroplets as enzyme active membrane-free protocells for detoxification of uric acid in serum. *Chemical Communications* **2019,** *55* (92), 13880-13883.
23. Jia, L.; Ji, Z.; Ji, Y.-m.; Zhou, C.; Xing, G.-w.; Qiao, Y., Design and Fluorescence Localization of Lipid-Rich Domains in Multiphase Coacervate Droplets Based on AIE-Active Molecules**. *ChemSystemsChem* **2021,** *3* (2), e2000044.
24. Williams, D. S.; Koga, S.; Hak, C. R. C.; Majrekar, A.; Patil, A. J.; Perriman, A. W.; Mann, S., Polymer/nucleotide droplets as bio-inspired functional micro-compartments. *Soft Matter* **2012,** *8* (22), 6004-6014.
25. Yin, Y.; Chang, H.; Jing, H.; Zhang, Z.; Yan, D.; Mann, S.; Liang, D., Electric field-induced circulation and vacuolization regulate enzyme reactions in coacervate-based protocells. *Soft Matter* **2018,** *14* (31), 6514-6520.
