# Supplementary figures and images for: Manipulation of coacervate droplets with an electric field
Source: Proc Natl Acad Sci U S A. 2022 Aug 4;119(32):e2203483119. doi: 10.1073/pnas.2203483119 (PMC9372540; doi:10.1073/pnas.2203483119)

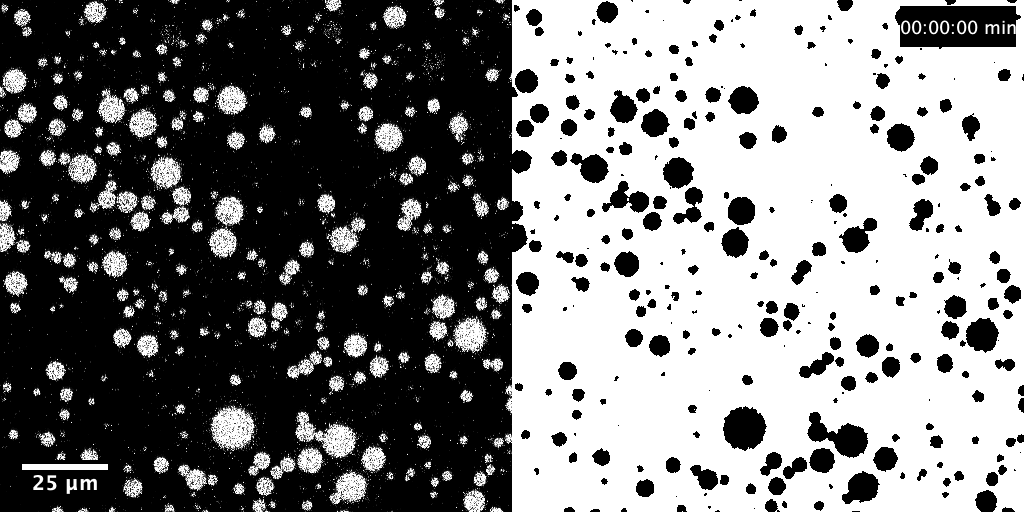

Supplement: Supplementary File [file pnas.2203483119.sm05.gif]
